# Does zinc with and without iron co-supplementation have effect on motor and mental development of children? A systematic review and meta-analysis

**DOI:** 10.1186/s12887-020-02340-1

**Published:** 2020-09-28

**Authors:** Firoozeh Sajedi, Soheila Shahshahani, Hesam Ghiasvand, Zahra Mosallanezhad, Shiva Fatollahierad

**Affiliations:** 1 Pediatric Neurorehabilitation Research Center, University of Social Welfare and Rehabilitation Sciences, Tehran, Iran; 2 Health Economics Group, Institute of Health Research, Medical School, University of Exeter, Exeter, UK; 3 Department of Physiotherapy, University of Social Welfare and Rehabilitation Sciences, Tehran, Iran; 4 Department of Global Public Health, Karolinska Institutet, Stockholm, Sweden

**Keywords:** Zinc, Iron, Child, Development

## Abstract

**Background:**

Effects of zinc with and without iron co-supplementation on child development are uncertain therefore the aims of this systematic review were to explore whether supplementation with zinc alone and zinc with iron in children aged 0–5 years old have beneficial or adverse effects on their mental and motor development.

**Method:**

We searched MEDLINE, EMBASE, Cochrane Central Register of Controlled Trials, CINAHL, Web of Science and Scopus until July 2020 and included randomized controlled trials, which assessed effects of zinc supplementation with and without iron in children less than 5 years old on mental and motor development. Data were pooled by random effects model and the Standardized Mean Differences (SMDs) with 95% confidence interval were estimated. The heterogeneity was assessed by I^2^.

**Results:**

Twenty-five studies with 11,559 participants were eligible to be included in this systematic review. Meta-analysis was conducted with eight articles that used Bayley Scales of Infant and Toddler Development II. We concluded that zinc alone and zinc with iron co-supplementation do not have beneficial or adverse effect on child mental and motor development at 6 and 12 months of age with low to moderate quality of the evidence. Furthermore, Zinc supplementation does not have any long term effect on child development in preschool and school age children.

**Conclusion:**

Most included studies did not show the efficacy of zinc with and without iron co-supplementation on child mental and motor development up to 9 years old age. Further Randomized Controlled Trials (RCTs) need to be taken into considerations the context-based differences between countries with special focus on socio-economic differences.

## Background

Child development is one of the most important aspects of pediatrics. The brain in early years of life is more vulnerable and has high plasticity [1]. Therefore, there is a pressing need to prevent developmental delay by conducting early interventions in infants and preschool children [2, 3]. Nutrition is one of the influential domains on child development [4, 5]. Zinc is a crucial micronutrient in the body which has high concentrations in synaptic vesicles of the glutamatergic neurons in the hippocampus and olfactory bulb [6]. It has also a significant role in DNA transcription, and subsequently brain development [7, 8].

The prevalence of zinc inadequate dietary intake varies between 7.5% in high income countries and 30% in South Asia. In Iran, 10.9% of 3–6 years old children were zinc deficient [9]. Zinc deficiency in 1–4 years old children in Mexico was 28.1% [10], and 59.09% of children less than 5 years old were zinc deficient in rural Nigeria. This difference of zinc deficiency prevalence could originates from varying consumption of animal-protein and high-phytate diets [11].

Exclusively breastfed infants can take required amount of zinc from breast milk in their first 6 months of life. However, after 6 months of age, the mother’s milk should be complemented with foods rich in zinc [12]. Apart from zinc rich foods, zinc fortification of foods and zinc supplementation can also increase zinc plasma concentration [13, 14]. Hence to reach zinc requirements, children with poor nutrition may need zinc supplementation [15].

The role of zinc in child development has been analyzed in several studies with mixed results. We have summarized the results of some systematic reviews in this regard. In a systematic review, Gogia et al. (2012) included 13 trials and identified that zinc had no significant effects on child development. Eight of the studies evaluated child development using Bayley Scales of Infant and Toddler Development (BSID). The results of their meta-analysis showed that the mean difference in Mental Development Index (MDI) and Psychomotor Developmental Index (PDI) was – 0.50 and 1.54 between zinc and placebo groups respectively at 12 month of age. The *p* values were insignificant with high levels of heterogeneity [16]. In another meta-analysis, Nissensohn et al. (2013) examined effects of zinc on MDI and PDI in 0–12 months old children. These authors also found that MDI and PDI were not significantly different in intervention and control groups [17].

Furthermore, some studies showed that zinc may decrease serum iron [18] and ferritin concentration [19] and Zinc co- supplementation with iron could interfere with absorption of both micronutrients [20, 21]. However some studies showed the beneficial effects of zinc with iron co-supplementation on child development [22, 23].

Effects of zinc supplementation on childhood development in 0–5 years old children were last assessed in 2012 however in this systematic review, we retrieved RCTs until July 2020 which supplemented children up to 5 years old and assessed their development in 0–5 years old and school- age. Therefore, we systematically reviewed the existing literature to address whether zinc alone and zinc co-supplementation with iron in children up to 5 years of age had any short or long term effects on child mental and motor development.

## Methods

### **Search strategy and selection criteria**

We retrieved the studies through searching the following databases and search engines: MEDLINE (Ovid), EMBASE (Ovid), Cochrane Central Register of Controlled Trials (CENTRAL), CINAHL (EBSCO), Web of Science and Scopus. References of included studies and previous related review articles were screened in order to identify other possible relevant studies. Databases of registered clinical trials including clinicaltrials.gov, WHO International Clinical Trials Registry Platform (ICTRP) and ISRCTN Registry were also screened. Furthermore, The American Journal of Clinical Nutrition was hand searched for other potential related articles. Medical Subject Headings (MeSH terms) and text words were used to search databases. The time span for searching was from inception initially to July 2017 and then updated to July 2020. We only included published studies with English abstracts. We used Google Translate to translate the non- English retrieved studies to English. Supplementary Table (1) depicts the search strategy in Ovid MEDLINE.

We included randomized controlled trials (RCTs) with randomization at either an individual or cluster level. The participants were children 0–5 years old at the time of supplementation without having HIV, developmental delay or developmental disorders such as autism, attention deficit hyperactivity disorder (ADHD), or intellectual disability. Furthermore, RCTs which supplemented children before 5 years of age but assessed them with developmental tests in school age were also included. The interventions of included studies were oral supplementation of zinc alone or zinc with iron, given on an intermittent or daily basis compared with either a placebo or no supplementation or with iron without zinc. We excluded studies that investigated food or formula milk fortification with zinc, zinc rich diet and parenteral zinc supplementation. Our primary outcome was the effect of zinc alone and in combination with iron supplementation in children 0–5 years of age on their mental and motor development in 0–5 year old and school- age.

This study employed Preferred Reporting Items for Systematic Reviews and Meta-Analyses (PRISMA) [24] to identify relevant articles and report the screening process. Two reviewers screened titles and abstracts of the articles for selecting relevant studies. Full text of potentially eligible articles meeting inclusion criteria were read by the two reviewers for inclusion in the review. Any disagreement about selecting an article was resolved through discussion. Data extraction of included studies was carried out by two review authors using a form designed for this review. Any discrepancies between the extracted data were discussed to reach a consensus. The extracted data were imported to Review Manager 5.3 by one reviewer.

For each study, we collected data on the following domains: author, publication date, study design, location and setting of the study, intervention date, sample size, age range, nutritional status, baseline length-for-age z-score, co morbidities, inclusion and exclusion criteria, zinc dosage, frequency of zinc supplementation, type of zinc compound, duration of the intervention, co-interventions, outcomes, outcomes assessments tools, results, method of allocation and randomization, blinding of participants and outcome assessors, exclusion of participants after randomization and proportion of losses to follow-up.

Details of methods in some sub- studies were extracted from the original article as the authors did not fully explain the methods [25–27]. In some cases, extra information was obtained by communicating with corresponding authors of articles. Furthermore, two reviewers independently evaluated the risk of bias for all included studies using the Cochrane Collaborations’ tool for assessing risk of bias in randomized trials [28, 29]. We resolved any disagreement by discussion. The risk of bias tool assesses the following criteria: Random sequence generation (checking for possible selection bias), allocation concealment (checking for possible selection bias), blinding of participants and personnel (checking for possible performance bias), blinding of outcome assessment (checking for possible detection bias), incomplete outcome data (checking for possible attrition bias through withdrawals, dropouts, protocol deviations), selective reporting (checking for possible reporting bias) and other sources of bias. The reviewers’ judgments were categorized as ‘Low risk’ of bias, ‘High risk’ of bias or ‘Unclear risk’ of bias. In addition, we applied GRADE criteria to assess the quality of evidence [30]. The GRADE Pro/GDT software was used to perform and illustrate the GRADE approach. We downgraded the high quality evidence by one level for serious concerns about risk of bias, inconsistency, indirectness, imprecision and publication bias criteria. The quality of each outcome is described as High, Moderate, and Low and very low based on these criteria. An I^2^ of more than 75% were considered high heterogeneity and on the condition that an outcome had high heterogeneity, the quality evidence was downgraded by one level. For determining the risk of bias of each outcome in a study, we defined three main domains in risk of bias tool. These domains were “random sequence generation”, “allocation concealment” and “blinding of outcome assessment”. If all three of them were low risk in a study, the outcome of that study was considered low risk. If one domain was unclear or high risk, the outcome of that study was considered unclear or high risk respectively. Finally, the risk of bias of each outcome between studies for GRADE quality was determined.

### **Data synthesis and statistical analysis**

We performed a meta-analysis on articles that used BSID second edition. Because of the considerable diversity in methods of assessing development; we excluded other articles that applied non-BSID II developmental screening tools from statistical analysis.

We ran random effects model in studies encompassed continuous outcomes. The reason for conducting the random effects approach was the high level of I square- which is the main statistics for assessing the heterogeneity- and clinical heterogeneity. Publication bias was assessed using Egger’s test and illustration with the funnel plot. We estimated the Standardized Mean Difference (SMD) with 95% confidence interval through Review Manager 5.3 [31] and Metan command in STATA 14. We conducted two main analyses. First we pooled data between zinc alone studies and the studies that had zinc alone arm in their multi arm interventions, to explore the effect of zinc without iron co-supplementation on child development. Second pooling data were between zinc co-supplementation with iron trials and the multi arm intervention studies that had zinc with iron arm. All these analyses were done at both 6 and 12 months of age assessment time. If a study report was not in these two time points, we considered their data in the nearest assessment time points. The data of 7 months assessment times were considered in 6 months time point and 10, 13 and 15 months’ assessment times in 12 months time point for meta-analysis.

The significance level for assessing these analyses was 0.05. As a result of small number of studies in each categorical variable, the sub-group analysis and sensitivity analysis were not performed. In addition, because of the small number of studies, it was not applicable to exclude articles with high risk of bias from meta-analysis.

## Results

### **Study selection**

**Fig. 1:**
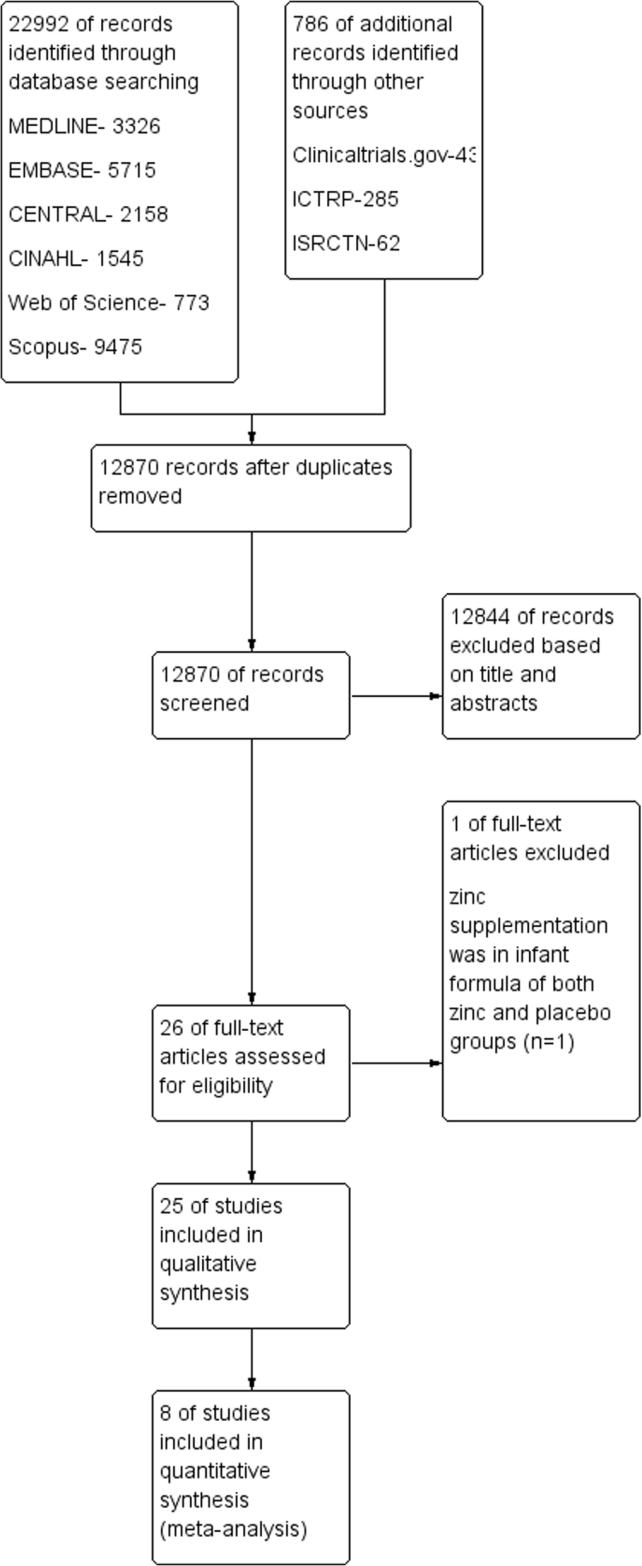
Study flow diagram

The search resulted in 22,992 records. After removing duplicate articles and performing the screening phase, 25 RCT studies with 11,559 participants were eligible to be included in the systematic review. The study flow diagram is illustrated in Fig. 1.>

### **Study characteristics**

The characteristics of studies are presented in Table 1

**Table 1:** Characteristic of the included studies

Study (year)	Research design; location	Participants	Baseline length-for-age Z-score	Intervention/Control groups	Zinc dosageFrequency	Zinc salt/Formulation	Intervention duration	Co-supplementation with Iron
Ashworth 1998 [32]	Randomized Controlled Trial; Brazil	Low birth weight term infants, low income families	Length at birth:placebo:45.9 ± 1.47,1 mg zinc group:46.2 ± 1.22,5 mg zinc group:46.5 ± 1.24	1 mg Zinc (*n* = 68), 5 mg Zinc (*n* = 71)/Placebo (*n* = 66)	1 mg or 5 mg ZincDaily except Sundays	Zinc sulfate/Syrup	eight weeks from birth	No
Bentley 1997 [33]	Randomized Controlled Trial; Guatemala	6–9 months old infants	N/A	Zinc (*n* = 43)/Placebo (*n* = 42)	10 mg ZincDaily	Zinc sulfate/ Syrup	7 months	No
Black Baqui 2004 [22]	Randomized Controlled Trial; Bangladesh	6 months old infants	Zinc group:- 1.2 ± 0.8,Iron group:- 1.2 ± 0.8,Iron+ zinc group:- 1.2 ± 0.7,Placebo group:- 1.2 ± 0.9	Iron+ Zinc (*n* = 74)/Iron (*n* = 72),Zinc (*n* = 70)/ Placebo (*n* = 65)	20 mg Zinc/ 20 mg IronWeekly	Zinc acetate/ Capsule	6 months	Yes in two arms of trial
Black Sazawal 2004 [34]	Randomized Controlled Trial; India	One month old, SGA infants	N/A (at 9 months old for zinc group: −1.8 ± 1.1, no zinc group: −2.0 ± 1.1)	a micronutrient mix including riboflavin, calcium, phosphorus, folate, and iron with zinc sulfate (*n* = 100)/ the same micronutrient mix without zinc (n = 100)	5 mg Zinc/ 10 mg IronDaily	Zinc sulfate/ Syrup	8 months	Yes
Castillo-Durán 2001 [35]	Randomized Controlled Trial; Chile	20 days old infants	N/A (at 6 months old for zinc group: − 0.06 ± 0.74, placebo group: − 0.06 ± 0.75)	Zinc (*n* = 75)/ placebo (n = 75)	5 mg ZincDaily	Zinc sulfate/N/A	12 months	No
Christian 2011 [36]	Cluster Randomized Controlled Trial; Nepal	7–9 years old children	height for age z score < − 2 (38.6–45.5%)	M-IFA C-IFAZn (*n* = 217) /M-IFA C-IFA (*n* = 164)M-IFAZn C-IFAZn (*n* = 124)/ M-IFAZn C-IFA (*n* = 137)	10 mg zincDaily	N/A/ Tablet	To age 36 months old	Yes
Colombo 2014 [37]	Randomized Controlled Trial; Peru	6 months old infants	Iron+copper+ zinc group:- 0.5 ± 0.9,Iron+copper group:- 0.6 ± 0.8	Iron + Copper + Zinc (*n* = 129) / Iron + Copper (122)	10 mg Zinc/10 mg ironDaily	Zinc sulfate/Syrup	12 months	Yes
Gardner 2005 [38]	Randomized Controlled Trial; Jamaica	9–30 months old, underweight children	Zinc groups:- 1.6 ± 0.82,Placebo groups:-1.25 ± 0.81	Zinc (*n* = 35)/zinc + stimulation (*n* = 26),placebo + stimulation (*n* = 23)/placebo (n = 42)	10 mg zincDaily	Zinc sulfate/ Syrup	6 months	No
Hamadani 2001 [39]	Randomized Controlled Trial; Bangladesh	less than 4 weeks old infants	Zinc group:- 1.1 ± 0.9,Placebo group:- 1.1 ± 0.8	Zinc (*n* = 104)/ Placebo (*n* = 109)	5 mg ZincDaily	Zinc acetate/ Syrup	5 months	No
Heinig 2006 [40]	Randomized Controlled Trial; United States of America	4 months old infants, fully breastfeed for ≥10 mo	Length in 4 months of age:Zinc group:64.2 ± 2.4,Placebo group:63.9 ± 2.4	Zinc (*n* = 41)/placebo (*n* = 44)	5 mg zincDaily	Zinc sulfate/Drop	182 days	No
Jimenez 2007 [41]	Randomized Controlled Trial; Cuba	one month old infants, low birth weight	N/A	Zinc (*n* = 87)/ Placebo (*n* = 76)	10 mg ZincDaily – divided to two doses in the first 6 month of life	Zinc sulfate/ Syrup	6 months	No
Katz 2010 [42]	Community-Based, Cluster Randomized, Placebo Controlled Trial; Nepal	1–35 months old children	N/A	Zinc (*n* = 759)/placebo (*n* = 847),Zinc + iron+ folic(*n* = 340) acid/iron+ folic acid (*n* = 242)	10 mg ZincDaily	Zinc sulfate/ Dispersible tablet	up to 36 months of age	Yes in two arms of trial
Lind 2004 [43]	Randomized Controlled Trial; Indonesia	6 months old infants, healthy singleton	Iron group: - 0.28 ± 0.81,Zinc group: - 0.33 ± 0.84,Iron+ zinc group: - 0.36 ± 0.83,Placebo group: - 0.41 ± 0.96	Iron+ Zinc (n = 170)/Iron (n = 170),Zinc (*n* = 170)/ Placebo (*n* = 170)	10 mg Zinc/ 10 mg IronDaily	Zinc sulfate/ Syrup	6 months	Yes in two arms of trial
Locks 2016 [44]	Randomized Controlled Trial; Tanzania	6 weeks old infants	Zinc groups:-0.43 ± 1.23,No zinc groups: −0.25 ± 1.16	Zinc (*n* = 62)/Placebo (*n* = 66),Zinc+ Multivitamin (*n* = 59)/Multivitamin (*n* = 60)	5 mg Zinc for 6 week to 6 months and 10 mg for 7–18 monthsDaily	Zinc sulfate/ Capsule	From ages 6 weeks to 18 months	No
Mathur 2015 [45]	Randomized Controlled Trial; N/A	Preterm neonates, Less than 7 days old, Exclusively breastfed during study period	N/A	Zinc (*n* = 50)/no placebo (*n* = 50)	2 mg/kg/ day	Zinc gluconate/syrup	untill 3 month of corrected age	No
Murray-Kolb 2012 [46]	Cluster Randomized Controlled Trial; Nepal	7–9 years old children	Iron, folic acid and Zinc group:- 1.93 ± 0.87,Iron and folic acid group:- 1.85 ± 0.91,Zinc group: - 1.97 ± 0.84,Placebo group:- 1.89 ± 0.90	Iron +Folic acid+ Zinc (*n* = 209)/ Iron+ Folic acid (*n* = 178);Zinc (*n* = 160) /Placebo(*n* = 188)	10 mg zincDaily	N/A/ Tablet	12 to 36 months of age	Yes in two arms of trial
Olney 2006 [47]	Community-Based, Randomized Controlled Trial; Tanzania (Pemba)	5–11 months old children	Iron+folic acid group: - 1.5 ± 1.0,Zinc group:- 1.3 ± 1.0 in,Iron+folic acid+zinc group:- 1.4 ± 1.2,Placebo group: - 1.6 ± 1.0	Zinc (*n* = 218)/Placebo(*n* = 215),Zinc + Iron+ Folic acid (*n* = 220)/Iron+ Folic acid (*n* = 223)	5 mg ZincDaily	N/A/ Dispersible tablet	One year	Yes in two arms of trial
Olney 2013 [23]	Community-Based, Randomized Controlled Trial; Tanzania (Pemba)	5–9 and 10–14 months old children	26% stunted in 5–9 months of age children36% stunted in 10–14 months of age children	Zinc/Placebo,Zinc + Iron+ Folic acid/Iron+ Folic acidTotal number = 528	10 mg Zinc more than 12 months of age and 5 mg Zinc in less than 12 months of age childrenDaily	N/A/ Dispersible tablet	One year	Yes in two arms of trial
Pongcharoen 2011 [48]	Randomized Controlled Trial; Thailand	9 years old children, breastfed at infancy	baseline length for age z score in original article:Zinc: - 0.9 ± 0.9,Iron: - 0.9 ± 0.9,Iron+ zinc group:- 0.8 ± 0.9,Placebo group: - 0.8 ± 0.9	Zinc (*n* = 139)/Placebo (*n* = 139)Zinc + Iron (*n* = 135)/Iron (*n* = 147)	10 mg Zinc10 mg Ferrous sulfateDaily	Zinc sulfate/Syrup	6 month	Yes in two arms of trial
Prado 2016 [49]	Cluster Randomized Controlled Trial; Burkina Faso	8.8 to 9.9 months of age	LNS-Zn0 group: −1.18 ± 1.08,LNS-Zn10 group: −1.31 ± 1.12,LNS-TabZn5 group:-1.07 ± 1.09	LNS-Zn10(*n* = 326)/ LNS-Zn0(*n* = 328)LNS-TabZn5(n = 326)/ LNS-Zn0(n = 328)	10 mg with LNSDaily	Zinc sulfate/ with LNS or tablet5 mg zinc in the form of tablet	9 months	Yes (6 mg iron in LNS product)
Sazawal 1996 [50]	Randomized Controlled Trial; India	children 6 to 35 months	N/A	Zinc (*n* = 48)/Placebo (*n* = 45)	10 mg ZincDaily	Zinc gluconate/ Syrup	1 to 6 months	No
Siegel 2011 [51]	Randomized Controlled Trial, Nepal	aged 53 weeks or less children	22% stunted	Zinc/placeboZinc + iron+ folic acid/iron+ folic acidTotal number = 259	5 mg zincDaily	Zinc sulfate/ Dispersible tablets	0–37 weeks	Yes in two arms of trial
Sudfeld 2019 [52]	Randomized Controlled Trial; Tanzania	6 weeks old	N/A	Zinc (*n* = 101)/placebo (*n* = 92)Zinc and multivitamins (n = 66)/ multivitamins (*n* = 106)	5 mg zinc less than 6 months of age10 mg zinc more than 6 months of ageDaily	Zinc/ Capsule	6 weeks to 18 months old	NO
Surkan 2013 [53]	Cluster Randomized Controlled Trial; Nepal	4–17 months old	N/A	Zinc (*n* = 127)/placebo (*n* = 152)Zinc + iron+ folic acid (*n* = 161)/iron+ folic acid (*n* = 129)	10 mg zincChildren less than one year old received 5 mg zincDaily	Zinc sulfate/ Dispersible tablets	One year	Yes in two arms of trial
Taneja 2005 [54]	Randomized Controlled Trial; India	12–18 months old infants	34.6% of zinc and 38.9% of placebo infants had length- for -age z -score less than −2 SD	Zinc (*n* = 327) /Placebo (*n* = 323)	10 mg Zinc20 mg Zinc older childrenDaily	Zinc gluconate/ Syrup	4 months	No

*N/A* the data is not available in the article

*M* Mother, *I* Iron, *F* Folic acid, *C* Child, *PL* Placebo, *Zn* Zinc

. Eleven studies only supplemented children with zinc alone [32, 33, 35, 38–41, 44, 45, 50, 54] and four studies co-supplemented them with zinc and iron [34, 36, 37, 49]. Ten trials had three parallel arms that one arm received zinc and iron; another arm received iron; and the third arm received zinc alone [22, 23, 42, 43, 46–48, 51–53].

With regard to child development assessment tools, nine studies assessed the child development by BSID second edition [22, 32, 34, 35, 37, 39, 41, 43, 54], one study evaluated the development by BSID third edition [44], and 15 studies used other developmental assessment tools [23, 33, 36, 38, 40, 42, 45–53]. Furthermore four studies were RCTs that supplemented children before the age of 5 years old and assessed their development in school age [36, 46, 48, 52]. The rest of the studies evaluated the development before the age of five. All of the included studies were in English however only one of the them was published in Spanish and Google Translate was used to translate it into English [41]. Table 2.

**Table 2:** Results of the included studies

Study (year)	Outcome	Assessment tool	Assessment time	Conclusion
Ashworth 1998 [32]	Development (motor, mental and behavior)	BSID-II	6 and 12 month of age	MDI: no difference PDI: no difference
Bentley 1997 [33]	Motor development	Time sampling observation method	Enrollment, 3 and 7 months of supplementation	Motor: no difference at 3 months follow up and better in zinc group at 7 months follow up(more time playing, sitting up and less time lying down and crying)
Black Baqui 2004 [22]	Development (motor, mental and behavior)	BSID-II	Baseline and 12 month of age	MDI: better in Iron+ zinc groupPDI: no difference
Black Sazawal 2004 [34]	Development (motor, mental and behavior)	BSID-II	6 and 10 months of age	MDI: no differencePDI: no difference
Castillo-Durán 2001 [35]	Development (motor and mental)	BSID-II	6 and 12 months of age	MDI: no differencePDI: no difference
Christian 2011 [36]	Motor (fine and gross) functioninggeneral intelligenceexecutive functioning	MABC and finger tapping testUNITStroop test, backward digit span, go/no-go tasks	7–9 years of age	Motor: no differenceMental: no difference
Colombo 2014 [37]	Development (motor and mental)	BSID-II	Baseline,12 and 18 months of age	MDI: no differencePDI: no difference
Gardner 2005 [38]	Development	4 subscales of the Griffiths Mental Development Scales	Enrollment and 6 months follow up	Hand and eye coordination: better in zinc group
Hamadani 2001 [39]	Development (motor and mental)	BSID-II	7 and 13 months of age	7 months assessment:MDI: no differencePDI: no difference13 months assessment:MDI: worse in zinc groupPDI: no difference
Heinig 2006 [40]	Motor development	Alberta Infant Motor Scale (AIMS)	4 (baseline) and 10 months of age	Gross motor development: no difference
Jimenez 2007 [41]	Development (motor and mental)	BSID-II	Baseline, 3, 6, 9 and 12 months of age	MDI: no differencePDI: better in zinc group at 6 months
Katz 2010 [42]	Age at first walking unassisted	Pictures of 14 sequential motor milestones/	Weekly interview with the child’s caregiver	Mean age at first walking unassisted: no difference
Lind 2004 [43]	Development (motor, mental and behavior)	BSID-II	Baseline and 12 months of age	MDI: no differencePDI: no difference
Locks 2016 [44]	Development (cognition, language, and motor)	BSID-III	15 months of age	No difference in any domains of BSID-III
Mathur 2015 [45]	Neurodevelopment	Amiel-Tison method	40 weeks conceptual age and 3 month corrected age	Attention span: better in zinc group at 40 weeks conceptual ageHyper-excitability: higher number in control group at 40 weeks conceptual age and 3 month corrected age
Murray-Kolb 2012 [46]	General intelligenceexecutive functioning	UNITStroop test, backward digit span, go/no-go tasks	7–9 years of age	Motor: no differenceMental: no difference
Olney 2006 [47]	Motor development (the time it took for children to walk unassisted)	picture chart containing 14 gross motor milestones based on the work of McGraw	Every two week for one year	Motor: no difference
Olney 2013 [23]	Development (motor, social emotional, exploratory behavior and language)	Picture chart containing 14 gross motor milestones	Every two week for one year	Gross motor: better in iron+ folic acid+ zinc in 5–9 months group and better in iron+ folic acid in 10–14 months groupMotor activity: better in iron+ folic acid+ zinc in 10–14 months group
Pongcharoen 2011 [48]	Cognitive performance	Wechsler Intelligence Scale for Children–Third edition (WISC-III; Thai version) and Raven’s Colored Progressive Matrices (CPM)	9 years old	Mental: no difference
Prado 2016 [49]	Development (motor, language, and personal-social development)	Developmental Milestones Checklist II	18 months of age	Motor: no difference
Sazawal 1996 [50]	Activity levels	Observation and recording	12 to 23 months of age	Activity level: better in zinc group
Siegel 2011 [51]	Cognitive development	Information-processing measures that were part of the FTII and the A-not-B Task	39 and 52 week old	Mental: no difference
Sudfeld 2019 [52]	Development	Koh’s Block Design testVerbal Fluency testEast African Neurodevelopment Tools	6–8 years old	General intelligence: no differenceExecutive function: no difference
Surkan 2013 [53]	Development (parental report of Motor and Language Milestones)	Motor and language milestone instruments were adaptedfrom the Griffiths Mental Development Scale and the MacArthur Communicative Development Inventory	Baseline and three month intervals for one year.	Motor: no difference
Taneja 2005 [54]	Development (motor and mental)	BSID-II	4 months after supplementation	MDI: no differencePDI: no difference

*N/A* the data is not available in the article

depicts the results of the studies. Supplementary File 1 shows the characteristics of all included studies more comprehensively.

### **Risk of bias in included studies**

**Fig. 2:**
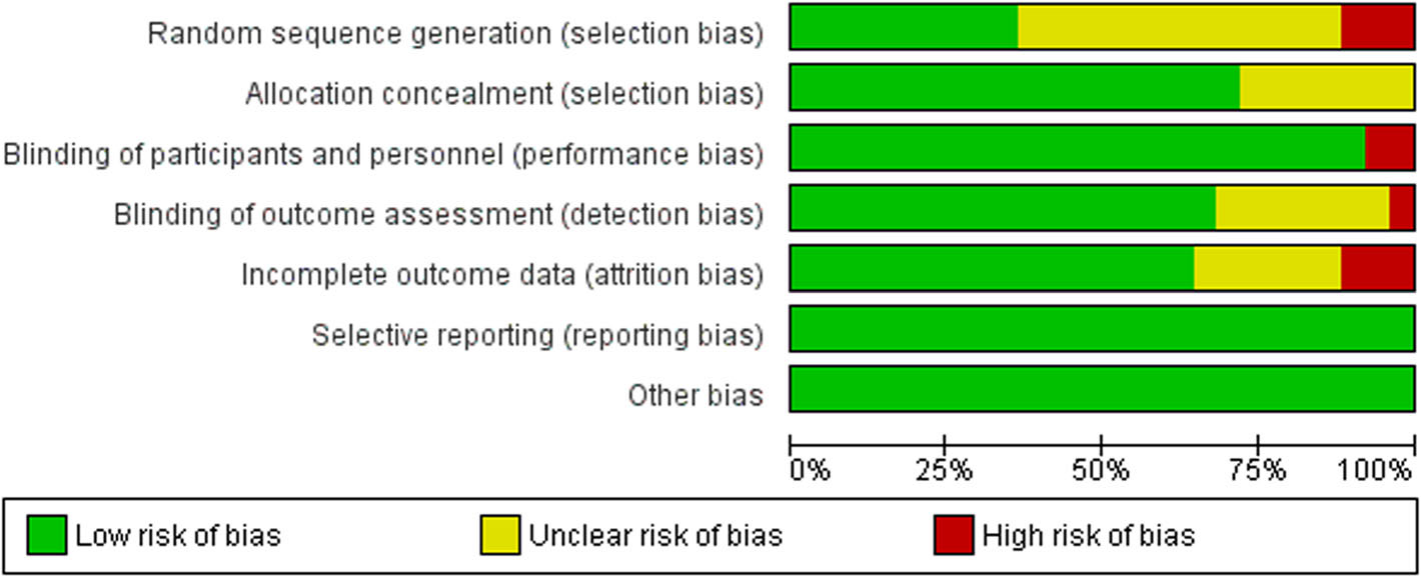
Risk of bias summary

Risk of bias of included studies was evaluated with Cochrane Risk of Bias Tool for Randomized Controlled Trials. In the random sequence generation domain, 13 articles had unclear risk [23, 33–39, 42, 46, 47, 50, 51], nine articles had low risk [40, 41, 43, 45, 48, 49, 52–54] and three studies had high risk of bias [22, 32, 44]. In addition, 18 articles had low risk of bias [23, 33–37, 40, 41, 43–48, 50–52, 54], seven studies had unclear risk of bias [22, 32, 38, 39, 42, 49, 53] and no study had high risk of bias in allocation concealment domain. Two articles had a high-risk of bias [45, 49] in blinding of participants and personnel domain and the rest of the articles had low risk of bias. Furthermore, 17 studies had low risk of bias [23, 32–34, 37–39, 42–44, 47–53], seven articles had unclear risk of bias [22, 35, 36, 40, 41, 46, 54], and one study had high risk of bias [45] in blinding of outcome assessment domain. Three studies had high risk of bias [22, 32, 42], six trials had unclear risk of bias [33, 35, 41, 49, 52, 54] and 16 articles had low risk of bias in incomplete outcome data [23, 34, 36–40, 43–48, 50, 51, 53]. Figures 2

**Fig. 3:**
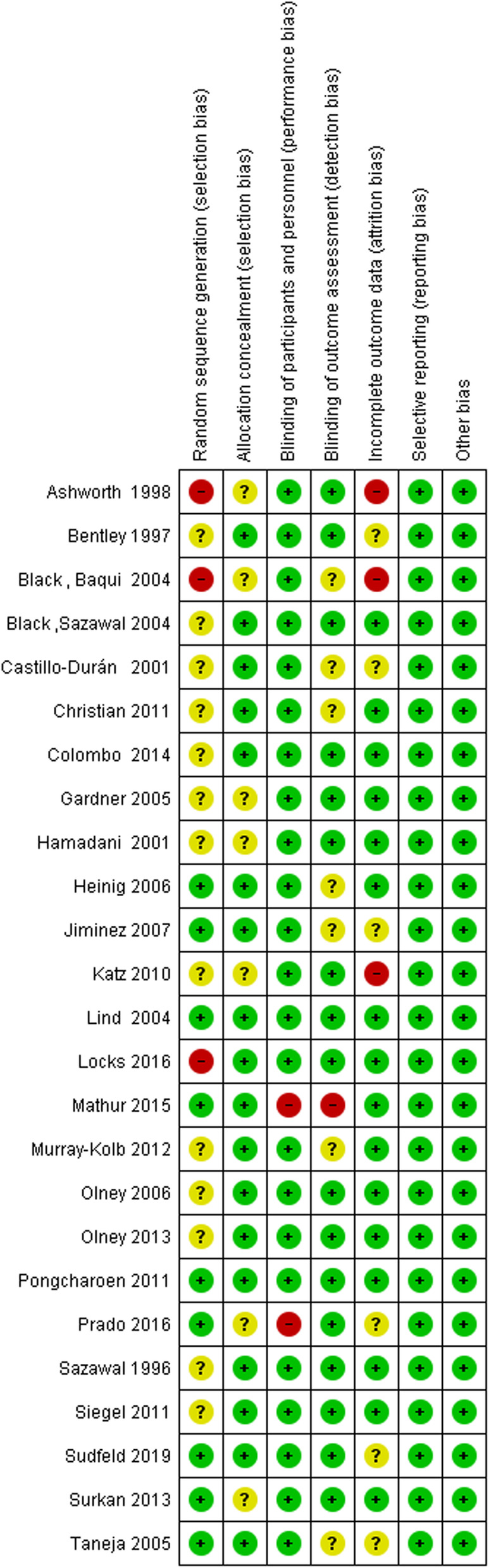
Risk of bias graph

and 3 show the risk of bias summary and graph respectively.

In addition, four studies had high risk of bias [22, 32, 44, 45], three studies had low risk of bias [43, 48, 52] and the rest of them had unclear risk of bias for developmental outcomes.

### **Meta-analysis findings**

Meta-analyses with 8 articles that used BSID second edition were performed. We excluded the study conducted by Taneja et al. from analysis since the duration of zinc supplementation intake was different among children [54]. We also excluded the 1 mg zinc arm of Ashworth et al. study from our analysis since the dosage used in their study was lower than the minimum 5 mg zinc dose used in the other studies. These Meta analyses assessed the effects of zinc on mental and motor domains of development at two time points (6 and 12 months old children). The developmental assessment times of most of the included studies were less than 12 months of age. Therefore, we could not analyze the developmental changes in children above one-year-old in meta-analysis.

Funnel plots for assessing publication bias had symmetrical appearances that are presented in supplementary Figures 1, 2, 3, 4, 5, 6, 7 and 8. Furthermore, the Egger’s test results for all outcomes in both two time points (6 and 12 months) were also not statistically significant (*P* > 0.05 for all of the slopes) and they are presented in details in supplementary appendix. Table 3 demonstrates the results of meta-analysis. Forest plots of meta-analyses are presented in Figs. 4, 5, 6, 7, 8, 9, 10 and 11.

**Table 3:** Meta-analysis results

Outcomes by time points of BSID assessments	Number of Trials	Sample Size	SMD (95% CI)	Heterogeneity Statistics
Zinc Alone vs. Placebo				
6 Months:				
MDI	4	591	-0.18 (−0.39 to 0.02)^a^	I^2^ = 37.4%, Χ^2^ = 4.79
PDI	4	591	0.17 (−0.20 to 0.55)	I^2^ = 80.8%, Χ^2^ = 15.60
12 Months:				
MDI	6	977	-0.08 (−0.36 to 0.19)	I^2^ = 77.4%, Χ^2^ = 22.14
PDI	6	977	0.30 (−0.24 to 0.83)	I^2^ = 94.00%, Χ^2^ = 80.23
Zinc with Iron vs. Iron				
6 Months:				
MDI	2	359	0.09 (−0.11 to 0.30)	I^2^ = 0.00, Χ^2^ = 0.01
PDI	2	359	0.07 (−0.14 to 0.28)	I^2^ = 0.00, Χ^2^ = 0.42
12 Months:				
MDI	4	790	-0.03 (−0.17 to 0.11)	I^2^ = 0.00, Χ^2^ = 1.02
PDI	4	790	0.01 (−0.24 to 0.26)	I^2^ = 66.40, Χ^2^ = 8.94

^a^ Significant at 10% level

**Fig. 4:**
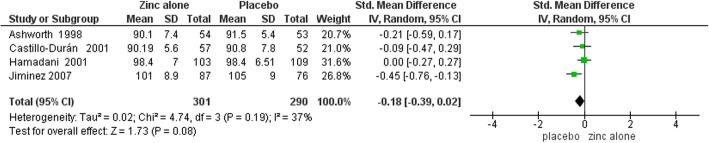
Forest plot of zinc alone versus placebo on MDI at 6 months of age

**Fig. 5:**
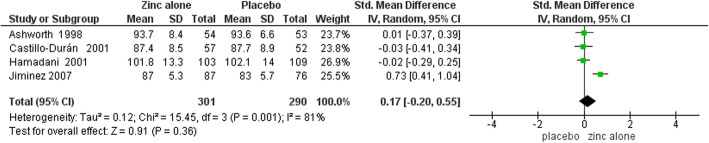
Forest plot of zinc alone versus placebo on PDI at 6 months of age

**Fig. 6:**
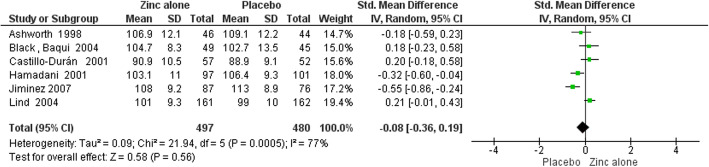
Forest plot of zinc alone versus placebo on MDI at 12 months of age

**Fig. 7:**
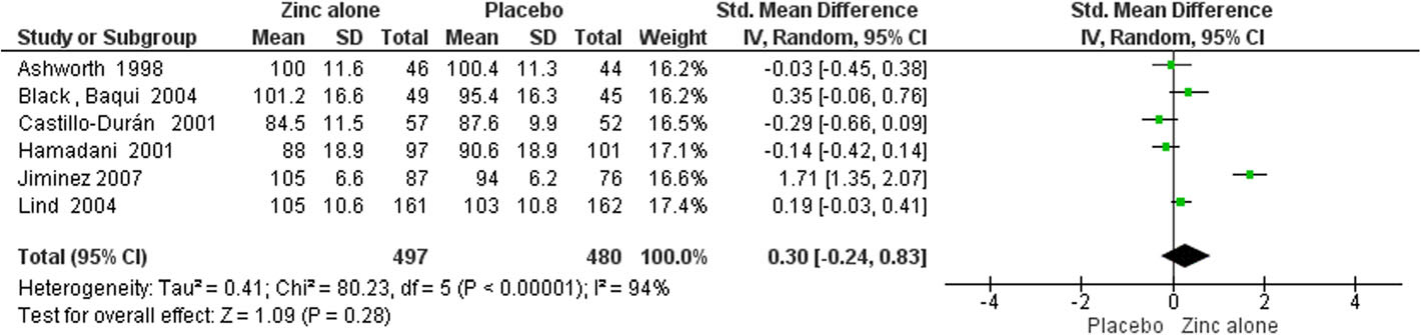
Forest plot of zinc alone versus placebo on PDI at 12 months of age

**Fig. 8:**
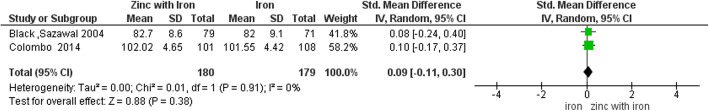
Forest plot of zinc with iron versus iron on MDI at 6 months of age

**Fig. 9:**
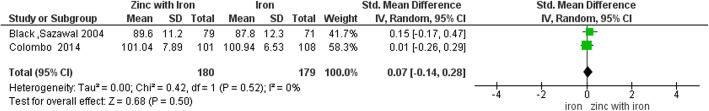
Forest plot of zinc with iron versus iron on PDI at 6 months of age

**Fig. 10:**
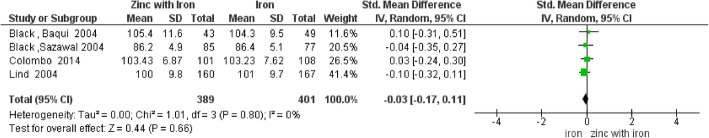
Forest plot of zinc with iron versus iron on MDI at 12 months of age

**Fig. 11:**
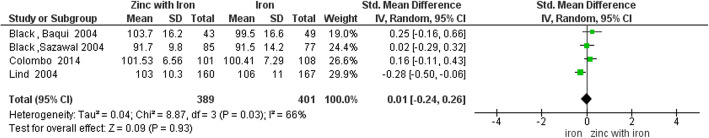
Forest plot of zinc with iron versus iron on PDI at 12 months of age

In meta-analysis of zinc alone versus placebo groups, four studies at 6 months assessment time and six trials in 12 months assessment time with BSID II were included. There is moderate quality evidence that MDI at 6 months of age was not different in zinc alone supplementation group compared with control group (SMD: -0.18, 95% CI: − 0.39 to 0.02, *p* value: 0.08, 591 participants). There is low quality evidence that zinc alone had no beneficial or negative effect on MDI at 12 months of age (SMD: -0.08, 95% CI: − 0.36to 0.19, *P* value: 0.56, 977 participants).

Zinc alone supplementation had no impact on PDI at 6 months of age (SMD: 0.17, 95% CI: −.0.20 to 0.55, *P* value: 0.36, 591participants) and at 12 months of age (SMD: 0.30, 95% CI: - 0.24 to 0.83, P value: 0.28, 977 participants) with low quality evidence.

The results of two studies at 6 months assessment time and four studies at 12 months assessment time with BSID II were pooled to assess the effect of zinc with iron co-supplementation versus iron. There is moderate quality evidence that zinc with iron co-supplementation compared with iron does not have beneficial or adverse effect on MDI at 6 months of age (SMD: 0.09, 95% CI: − 0.11 to 0.30, P value: 0.38, 359 participants), PDI at 6 months of age (SMD: 0.07, 95% CI: − 0.14to 0.28, P value: 0.50, 359 participants), MDI at 12 months of age (SMD: -0.03, 95% CI: − 0.17 to 0.11, P value: 0.66, 790 participants) and PDI at 12 months of age (SMD: 0.01, 95% CI: − 0.24 to 0.26, P value:0.93, 790 participants.).

### **Qualitative analysis findings**

Seventeen studies data were not in meta-analysis and their results are summarized in Table 2.

In Bentley et al. study in 1997, motor development was assessed with observational method at 3 and 7 months of supplementation. The motor development of zinc group was better at 13 to 16 months of age however there was no difference in 9 to 12 months old [33]. In Gardner et al. study, 6 months supplementation with zinc in underweight children of 9–30 months old increased their hand and eye coordination [38]. Motor development at 10 months of age with daily zinc was not different in intervention and control group in Heinig et al. study [40]. In Katz et al. study, zinc with and without iron had no effect on mean age at first walking unassisted [42]. Locks et al. assessed child development with BSID third edition and showed that zinc supplementation had no effect on any domain of development at 15 months of age [44] .

In Mathur et al. study, attention span was better in zinc group at 40 weeks and higher number of excitability in control group at 40 weeks and 3 months old [45]. In Olney et al. study in 2006, zinc with and without iron supplementation in children 5–11 months of age had no effect on the time of unassisted walking. In Olney et al. study in 2013, effect of zinc with and without iron co supplementation in children 5–9 months old and 10–14 months old were assessed. Gross motor development at 5–9 months old and motor activity at 10–14 months old groups in zinc with iron co supplementation was better than control groups. Olney study in 2006 and Olney study in 2013 are two sub studies of Sazawal et al. study in 2006 [27]. In Prado et al. study, there was no difference in zinc and control group in motor development at 18 months of age [49]. In Sazawal et al. study, activity level were better in zinc group at 12 to 23 months old [50]. In Siegel et al. study, Zinc with and without iron had no effect on mental development at 39 and 52 weeks old [51]. In Surkan et al. study, zinc with and without iron had no effect on child motor development in 1 year supplementation in 4–17 months old infants [53]. Taneja et al. showed that 4 months supplementation with zinc alone had no effect in mental and motor development of children at 16–22 months of age [54].

Four studies assessed the long term effect of zinc supplementation with and without iron on child development in 6–9 year old children. All of them were supplemented before 36 months old [36, 46, 48, 52]. These studies showed that zinc supplementation with and without iron does not have any long term effect on child mental and motor development in school age. Christian et al. study showed that supplementation with zinc and iron up to 36 months of age had no developmental benefit at 7–9 year old children [36]. Murray-Kolb showed that supplementation with zinc alone or zinc and iron in 12 to 36 months old children do not have long term effect on 7–9 years old children mental and motor development. Murray- Kolb 2012 [46] and Katz 2010 [42] are from one original study in Nepal. Pongcharoen et al. study also showed that supplemented with zinc with and without iron for 6 months in 4–6 months old infants had no effect on their mental development at 9 years old [48]. Sudfeld et al. study is the follow up study of Locks study at 6–8 years old. They also showed that zinc supplementation up to 18 months of age had no effect on child development at school-age [52].

### **Zinc effect in low and middle income countries**

Reviewing the final included studies in our study, we found that 23 of the trials were conducted in low and middle income countries. And two studies were carried out in high income countries. Four trials in low and middle income countries showed the efficacy of zinc alone on mental and/or motor development of children [33, 38, 41, 50] In addition, two studies in these countries showed positive effect of zinc with iron supplementation on child mental and/or motor development [22, 23]. But the rest of the studies and trials in high income countries did not show any beneficial effects of zinc with and without iron supplementation on child development.

## Discussion

In this systematic review and meta- analysis, we tried to answer, whether zinc alone or zinc with iron co-supplementation in children 0–5 year old have any short or long term effect on child mental and motor development. It is possible that iron and zinc have molecular interactions with each other and zinc alone and zinc with iron co-supplementation have different effects on the children’s developing body. It is also possible that zinc with iron could be better for development of children with lower z-score in the growth chart and children with malnutrition. Thus, we analyzed zinc alone and zinc with iron co-supplementation effect on child development in this systematic review and meta-analysis, separately.

The 6 and 12 months time points were chosen for meta- analysis, based on available data and with the consideration that exclusively breastfed infants may not benefit from zinc on 6 months of age because of enough intake of needed zinc from breast milk but infants may benefit from zinc supplementation at 12 months of age.

### **Zinc alone supplementation effect on child development**

Twenty-one studies compared the effects of zinc alone with placebo on child development. Ten of them were multi arm intervention studies which we considered the zinc alone and placebo arms to be included in zinc alone assessment. One study showed beneficial effects of zinc on the child mental development [45], and four studies demonstrated the favorable effect of zinc supplementation on motor domain of child development [33, 38, 41, 50]. In addition, in one single study, zinc had adverse effect on mental development at 13 months of age [39]. In the rest of the studies, zinc had no statistically significant effect on child development. MDI and PDI in zinc alone group at 6 and 12 months of age did not have statistically significant results.

### **Zinc co-supplementation with iron effect on child development**

Thirteen studies analyzed zinc with iron co-supplementation effect on child development. Nine of them were multi arm studies. We considered zinc with iron arm as intervention group and iron arm as their control group. In one study mental development was better in zinc with iron co-supplementation [22] compared to control group whereas in another study zinc with iron had more positive effects on motor development [23]. In the rest of the studies, zinc with iron compared with iron had no statistically significant effect on child development. Comparisons of MDI and PDI in zinc with iron co-supplementation versus iron at 6 and 12 months of age did not have statistically significant results.

### **Zinc supplementation effect on child development in school-age**

Christian, Murray-Kolb, Sudfeld and Pongcharoen and their colleagues, studied the effect of zinc intake with and without iron before 5 years of age on children development in 6–9 years old [36, 46, 48, 52]. They did not find any developmental difference in intervention and control groups. More long-term studies are needed to evaluate the impact of zinc with and without iron on older children.

### **Quality of the evidence**

Using GRADE, we evaluated the certainty of the evidence to be moderate to low for described outcomes at 6 and 12 months of age in meta-analysis. The reasons for these judgments are outlined in GRADE certainty assessment Tables 2 and 3 in supplements.

Therefore, high quality RCTs are needed to confirm that zinc with and without iron have any positive or negative impact on child motor and mental development in children less than 1 year old.

### **Limitations of the review**

The primary outcomes were to assess the effect of zinc with and without iron co-supplementation in children less than 5 years old on their short and long term mental and motor development however the data were not similar enough to do meta-analyses in children above 1 year old. So, the long term effect of zinc was not assessed in meta-analysis on child development and the results of 17 studies were reported descriptively.

To our best knowledge, the contextual influencing factors on association of zinc alone or iron supplementation with zinc on the children development are expectedly need to be addressed through sub-group and sensitivity analysis. These types of additional analysis surely lead to better understanding of those associations. But in our meta-analysis due to considerable differences in studies variables, we were not able to perform further analysis in this aspect. Of course we can assume any interpretation of the results should be accompanied with cautions, but in a general viewpoint we cannot confirm the positive impact of zinc alone or iron supplemented by zinc on children development.

## Conclusion

In conclusion, no significant positive or negative effects on child mental and motor development were seen in zinc supplementation with or without iron groups compared with control groups at 6 and 12 months of age in Meta-analysis. Long term effects of zinc supplementation in children above 1 year old were not analyzed because of heterogeneity of outcome assessment tools. However, most of the studies showed that zinc with and without iron co-supplementation in children 0–5 year old had no impact on child short and long term development up to 9 years old.

## Supplementary information

**Additional file 1.** Characteristics and risk of bias tables of all included studies.

**Additional file 2: Table S1.** MEDLINE (Ovid) search strategy. **Table S2.** GRADE assessments for zinc alone comparisons. **Table S3.** GRADE assessments for zinc with iron comparisons. **Figure S1.** Funnel plot of comparison Zinc alone versus Placebo, outcome: MDI at 6 months of age. **Figure S2.** Funnel plot of comparison Zinc alone versus Placebo, outcome: PDI at 6 months of age. **Figure S3.** Funnel plot of comparison Zinc alone versus Placebo, outcome: MDI at 12 months of age. **Figure S4.** Funnel plot of comparison Zinc alone versus Placebo, outcome: PDI at 12 months of age. **Figure S5.** Funnel plot of comparison Zinc with Iron versus Iron, outcome: MDI at 6 months of age. **Figure S6.** Funnel plot of comparison Zinc with Iron versus Iron, outcome: PDI at 6 months of age. **Figure S7.** Funnel plot of comparison: Zinc with Iron versus Iron, outcome: MDI at 12 months of age. **Figure S8.** Funnel plot of comparison: Zinc with Iron versus Iron, outcome: PDI at 12 months of age.

## Data Availability

The datasets analyzed during the current study are not public, but are available from the corresponding author on reasonable request.

## Abbreviations

BSID: Bayley Scales of Infant Development
GRADE: Grading quality of evidence and strength of recommendations
MDI: Mental Developmental Index
MESH: Medical subject headings
PDI: Psychomotor Developmental Index
PRISMA: Preferred Reporting Items for Systematic Reviews and Meta-Analyses
RCT: Randomized controlled trials
SMD: Standardized Mean Difference
