# Supplementary material for: Does zinc with and without iron co-supplementation have effect on motor and mental development of children? A systematic review and meta-analysis
Source: BMC Pediatr. 2020 Sep 28;20:451. doi: 10.1186/s12887-020-02340-1 (PMC7520965; doi:10.1186/s12887-020-02340-1)
Supplement: Supplementary file 1 — Additional file 1. Characteristics and risk of bias tables of all included studies. [file 12887_2020_2340_MOESM1_ESM.pdf]

Characteristics of studies

Characteristics of included studies

Ashworth 1998

**Methods**	Partially Randomized Controlled Trial
**Participants**	Country:Brazil , setting:city<br>Intervention date: during a 13-month period beginning in January 1993, all singleton LBW term infants weighing 1500±2499 g born to families of low income (equivalent to <US$280 per month) were recruited for the study. During February-August 1994, 71 LBW term infants received 5 mg zinc.<br>Nutritional status:low income<br>Baseline length for age z score: N/A (length at birth or baseline of study was placebo:45.9±1.47, 1 mg zinc:46.2±1.22 and 5 mg zinc:46.5±1.24)<br>Inclusion criteria:Low birth weight term infants in ow income families<br>Exclusion criteria:Infants with congenital anomalies, abnormal neurological signs, or signs of asphyxia.<br>Number randomized:205
**Interventions**	Intervention group 1:<br>zinc (1 mg zinc)<br>number randomized:68 ; number analyzed at 6 month:56 ; number analyzed at 12 month:48<br>Intervention group 2:<br>zinc (5 mg zinc)<br>number randomized:71 ; number analyzed at 6 month:54 ; number analyzed at 12 month: 46<br>Control group:<br>placebo (sorbitol and flavour)<br>number randomized:66 ; number analyzed at 6 month:53 ; number analyzed at 12 month: 44<br>Formulation:syrup,zinc as zinc sulphate<br>Frequency of supplementation: daily except Sundays<br>Intervention duration:8 weeks from birth
**Outcomes**	Development (motor, mental and behavior)<br>Assessment tool: The Bayley Scales of Infant Development, version II (Bayley II)<br>Assessment time: 6 and 12 month of age
**Title**	Zinc supplementation, mental development and behaviour in low birth weight term infants in northeast Brazil
**Results**	"At 6 and 12-months, mental and psychomotor development was assessed with the Bayley Scales of Infant Development and no significant differences in the scores of the three groups were found."

**Correspondence address**	A Ashworth<br>Centre for Human Nutrition, London School of Hygiene and Tropical Medicine, Keppel Street, London WC1E 7HT
**Notes**	Quote:"The intention was to give 5 mg zinc or a placebo daily except Sundays, for eight weeks from birth, in a randomized, double-blind trial. A mistake in the manufacture of the zinc solution resulted in the initial cohort being given 1 mg instead of 5 mg. When this was discovered, the design was modified and the enrolment period was extended to include a second cohort of infants who would all receive 5 mg zinc. All field workers and participating families were unaware of the change and remained blind to treatment allocation."<br>"At 4, 8, 17 and 26 weeks, any zinc-containing ointments, creams or tonics given by parents were recorded. Consumption of specific zinc-rich foods was also ascertained on these occasions"

Risk of bias table

Bias	Authors' judgement	Support for judgement
Random sequence generation (selection bias)	High risk	Quote: "The 5 mg zinc group, however was not randomly allocated."
Allocation concealment (selection bias)	Unclear risk	No relevant quote found in article
Blinding of participants and personnel (performance bias)	Low risk	Quote: "All field workers and participating families were unaware of the change and remained blind to treatment allocation."
Blinding of outcome assessment (detection bias)	Low risk	Quote: "Two paediatricians shared the testing at 6-months, and one of them undertook all the 12-month tests. Both were blind to treatment allocation"
Incomplete outcome data (attrition bias)	High risk	Attrition at 6 month:20.5%<br>Attrition at 12 month:32.7%<br>Quote: "Examination of the characteristics of infants lost to follow-up showed that these were comparable across the three treatment groups"
Selective reporting (reporting bias)	Low risk	All outcomes specified in the methods were reported
Other bias	Low risk	No evidence of other bias

Bentley 1997

**Methods**	Randomized Controlled Trial
**Participants**	Country:Guatemala , setting:rural<br>Intervention date:N/A<br>Nutritional status:N/A<br>Baseline length for age z score:- 2.0±1 in zinc group and - 2.1±1.1 in placebo group

	Inclusion criteria:6-9 months old children<br>Exclusion criteria:none<br>Number randomized:108
**Interventions**	Intervention group :<br>zinc (10 mg zinc)<br>number analyzed:43<br>Control group:<br>placebo<br>number analyzed:42<br>Formulation:syrup, zinc as zinc sulfate<br>Frequency of supplementation:daily<br>Intervention duration:7 months
**Outcomes**	Motor development (Infant activity)<br>Assessment tool:time sampling-observation method<br>Assessment time:At enrollment, and after 3 and 7 months of supplementation
**Title**	Zinc Supplementation Affects the Activity Patterns of Rural Guatemalan Infants
**Results**	"No differences in motor development were observed by treatment group. However, at follow-up 2 (after 7 mo of supplementation), zinc-supplemented infants were significantly more frequently observed sitting up compared with lying down than unsupplemented infants."
**Correspondence address**	Margaret E. Bentley<br>The Johns Hopkins University, School of Hygiene and Public Health, Center for Human Nutrition, Baltimore,<br>MD 21205
**Notes**	

Risk of bias table

Bias	Authors' judgement	Support for judgement
Random sequence generation (selection bias)	Unclear risk	No relevant quote found in article
Allocation concealment (selection bias)	Low risk	Quote:"The supplements were indistinguishable, and neither the families nor the study staff were aware of the treatment group to which the infants belonged."
Blinding of participants and personnel (performance bias)	Low risk	Quote:"The supplements were indistinguishable, and neither the families nor the study staff were aware of the treatment group to which the infants belonged."
Blinding of outcome assessment (detection bias)	Low risk	Quote: "Data collectors were unaware of the household randomization"

Incomplete outcome data (attrition bias)	Unclear risk	attrition bias:21.3%<br>Quote : "There were 19 point. Analysis of co-variance techniques were then used to examine drop-outs, due to migration out of the village, mothers' work constraints or refusal. Two children did not complete the study because<br>of late enrollment, and two more children were found to have unreliable data and are not included in the analyses."
Selective reporting (reporting bias)	Low risk	All outcomes specified in the methods were reported
Other bias	Low risk	No evidence of other bias

Black , Baqui 2004

**Methods**	Randomized Controlled Trial (substudy)
**Participants**	Country: Bangladesh, setting: rural<br>Intervention date: N/A<br>Nutritional status:poor<br>Baseline length for age z score: - 1.2±0.8 in Iron, Zinc and multiple micronutrient groups,- 1.2±0.7 in Iron+ zinc group and - 1.2±0.9 in placebo group<br>Inclusion criteria:Infants were eligible if they were 6 mo of age, did not receive infant formula, were not severely malnourished (mid upper arm circumference ≥ 110 mm), were not severely anemic (hemoglobin ≥ 90 g/L), and did not have obvious neurologic disorders, physical disabilities, or chronic illnesses.<br>Exclusion criteria:none<br>Number randomized:346 infants
**Interventions**	Intervention group1 :<br>Iron (20 mg elemental iron and 1mg riboflavin)<br>number randomized:72; number analyzed:49<br>Intervention group 2:<br>Zinc (20mg elemental zinc and 1mg riboflavin)<br>number randomized:70; number analyzed:49<br>Intervention group 3:<br>Iron+ zinc (20 mg elemental iron, 20 mg elemental zinc, and 1 mg riboflavin)<br>number randomized:74; number analyzed:43<br>intervention group4 :<br>multiple micronutrient (2 times the recommended dietary allowance (based on World Health Organization standards) of thiamine, niacin, folic acid, pantothenic acid, iodine, copper, manganese, selenium, and vitamins C, D, E, B-6, and B-12 in addition to 20 mg elemental Fe, 20 mg elemental Zn, and 1 mg riboflavin.)<br>number randomized:65; number analyzed:35<br>Control group:<br>placebo (1 mg riboflavin)<br>number randomized:65; number analyzed:45<br>Formulation:capsules which were mixed with flavored syrup and fed to the infants, iron as ferrous sulfate, Zinc as zinc acetate

	Frequency of supplementation:weekly<br>Intervention duration:6 month from 6 to 12 month
**Outcomes**	Development (motor, mental, and behavior)<br>Assessment tool: The Bayley Scales of Infant Development, version II (Bayley II)<br>Assessment time:6 and 12 month of age
**Title**	Iron and zinc supplementation promote motor development and exploratory behavior among Bangladeshi infants
**Results**	"Iron and zinc administered together and with other micronutrients had a beneficial effect on infant motor development."
**Correspondence address**	MM Black, Department of Pediatrics, University of Maryland School of Medicine, 655 West Lombard Street, Suite 311, Baltimore, MD 21201. E-mail: mblack@peds.umaryland.edu.
**Notes**	

Risk of bias table

**Bias**	**Authors' judgement**	**Support for judgement**
Random sequence generation (selection bias)	High risk	Quote: "Every alternate infant was invited to participate in the developmental substudy"
Allocation concealment (selection bias)	Unclear risk	No relevant quote found in article
Blinding of participants and personnel (performance bias)	Low risk	Quote: "The mixtures were similar in taste and appearance"
Blinding of outcome assessment (detection bias)	Unclear risk	No relevant quote found in article
Incomplete outcome data (attrition bias)	High risk	Attrition:36.1%<br>Quote: "No differential dropout was observed across supplementation groups, sex, maternal education, anemia, growth measures at 6 mo, or performance on the Bayley II Scales at 6 mo."
Selective reporting (reporting bias)	Low risk	All outcomes specified in the methods were reported
Other bias	Low risk	No evidence of other bias

Black ,Sazawal 2004

**Methods**	Randomized Controlled Trial (substudy)
**Participants**	Country: India , setting:urban<br>Intervention date:between April 18, 1996, and November 7, 1998.<br>Nutritional status:Very low-income<br>Baseline length for age z score: N/A (at 9 month of age for zinc group : -1.8±1.1

	and for no zinc group -2.0±1.1)<br>Inclusion criteria:gestational age was >36 weeks;birth weight was below the 10th percentile for gestational age;no congenital problems, disabilities, or severe illnesses.<br>Exclusion criteria:none<br>Number randomized:200
**Interventions**	Intervention group :<br>micronutrient mix + zinc (riboflavin, calcium, phosphorus, folate, and iron with 5 mg of zinc sulfate)<br>number randomized:100; number analyzed at 6 month:79; number analyzed at 10 month:71<br>Control group:<br>micronutrient mix<br>(riboflavin, calcium, phosphorus, folate, and iron)<br>number randomized:100; number analyzed at 6 month:85; number analyzed at 10 month:77<br>Formulation: syrup,zinc as zinc sulfate<br>Frequency of supplementation:daily<br>Intervention duration:eight month from 30 days to 9 months of age
**Outcomes**	Development (motor, mental and behavior)<br>Assessment tool: The Bayley Scales of Infant Development, version II (Bayley II)<br>Assessment time:6 and 10 month of age
**Title**	Cognitive and Motor Development Among Small-for-Gestational- Age Infants: Impact of Zinc Supplementation, Birth Weight, and Caregiving Practices
**Results**	"There were no direct effects of zinc supplementation on the infants’ development at either 6 or 10 months"
**Correspondence address**	Department of Pediatrics, University of Maryland School of Medicine, 655 W Lombard St, Ste 311,<br>Baltimore, MD 21201. mblack@peds.umaryland.edu.
**Notes**	

Risk of bias table

**Bias**	**Authors' judgement**	**Support for judgement**
Random sequence generation (selection bias)	Unclear risk	Quote:"The randomization charts were designed to select 200 infants (100 each from groups 1 and 2) for the developmental substudy."
Allocation concealment (selection bias)	Low risk	Quote:"The randomization procedure was designed to ensure that no members of the field or evaluation teams were aware of group assignment."

Blinding of participants and personnel (performance bias)	Low risk	Quote:"The micronutrient preparations were identical in taste, consistency, appearance, and acceptability."<br>"The randomization procedure was designed to ensure that no members of the field or evaluation teams were aware of group assignment."
Blinding of outcome assessment (detection bias)	Low risk	Quote:<br>"The randomization procedure was designed to ensure that no members of the field or evaluation teams were aware of group assignment."<br>"The examiners were not aware of the infants' supplementation status, anthropometry, or home environment."
Incomplete outcome data (attrition bias)	Low risk	Attrition bias: 7% at 6 month and 19% at 10 month evaluation .<br>Quote:"Infants who were retained in the sample did not differ from those who were lost to follow-up on birth weight, ponderal index, gestational age, maternal education, or paternal education."
Selective reporting (reporting bias)	Low risk	All outcomes specified in the methods were reported
Other bias	Low risk	No evidence of other bias

Castillo-Durán 2001

**Methods**	Randomized Controlled Trial
**Participants**	Country:Chile , setting:urban<br>slum in Santiago, Chile<br>Intervention date:N/A<br>Nutritional status:low socioeconomic status<br>baseline length for age z score: N/A (At 6 months z scores zinc group: −0.06 ± 0.74, placebo group: −0.06 ± 0.75)<br>Inclusion criteria: term (≥ 37 weeks) singleton;birth weight >2300 g (appropriate for gestational age), no evidence of toxoplasmosis, rubella, herpes, cytomegalovirus, or Chagas disease, fetal alcohol syndrome, or congenital malformations that affect growth. Mothers had to be literate and without a history of drug abuse.<br>Exclusion criteria:<br>Infants were monitored for anemia at 6 months, and those with hemoglobin <10.5 g/dL were excluded. Infants whose monthly zinc intake was <50% of the prescribed volume were excluded.<br>Number randomized:150
**Interventions**	Intervention group :<br>zinc (5 mg zinc) before 20 days of age.<br>number randomized:75; number analyzed:57<br>Control group:<br>placebo (an equivalent dose of lactose)<br>number randomized:75; number analyzed:55

	formulation:zinc as zinc sulfate<br>frequency of supplementation:once daily<br>intervention duration:12 month
**Outcomes**	Development (motor and mental)<br>assessment tool: Bayley II Scale<br>assessment time: at 6 and 12 months of age
**Title**	Effect of zinc supplementation on development and growth of Chilean infants
**Results**	"Zinc supplementation may have a beneficial effect on mental development of healthy term infants."
**Correspondence address**	Carlos Castillo-Durán, MD, Instituto de Nutrición y Tecnología de los Alimentos,Universidad de Chile, Macul 5540, Santiago, Chile
**Notes**	All infants received iron (sulfate) drops, 1 to 2 mg/kg/d, after 5 months of age.

Risk of bias table

**Bias**	**Authors' judgement**	**Support for judgement**
Random sequence generation (selection bias)	Unclear risk	No relevant quote found in article
Allocation concealment (selection bias)	Low risk	Quote:"Codes were kept secret"
Blinding of participants and personnel (performance bias)	Low risk	Quote:a double-blind fashion
Blinding of outcome assessment (detection bias)	Unclear risk	No relevant quote found in article
Incomplete outcome data (attrition bias)	Unclear risk	Attrition: 25.3%
Selective reporting (reporting bias)	Low risk	Hemoglobin and cranial circumference are not reported however they are not related to developmental outcomes.
Other bias	Low risk	No evidence of other bias

Christian 2011

**Methods**	Cluster Randomized Controlled Trial (follow up)
**Participants**	Country: Nepal, Setting: rural<br>Intervention date: 2001 to 2005 (12 to 36 month old)<br>Assessment date: June 2007 to April 2009 (follow up at 7-9 year old children)<br>Nutritional status:height for age z score< -2 (38.6% - 45.5%)<br>Inclusion criteria:All children aged 1-35 months and living in households in the study area during<br>the baseline enrolment round were eligible<br>Exclusion criteria:none

**Interventions**	Intervention group 1:<br>M-IFA C-PL (referent group)<br>number enrollment:103 ; number analyzed:101<br>Intervention group 2:<br>M-IFA C-IFA<br>number enrollment:164; number analyzed:163<br>Intervention group 3:<br>M-IFA C-IFAZn<br>number enrollment:217 ; number analyzed:212<br>Intervention group 4:<br>M-IFAZn C-IFA<br>number enrollment:137 ; number analyzed:137<br>Intervention group 5:<br>M-IFAZn C-IFAZn<br>number enrollment:124 ; number analyzed:122<br>(M-IFA C-IFAZn versus M-IFA C-IFA<br>M-IFAZn C-IFAZn versus M-IFAZn C-IFA)<br>Formulation:dispersible tablets, iron as N/A, , zinc as N/A<br>Frequency of supplementation: one tablet daily (or half a tablet if < 1 year old)<br>M= mother, I:iron, F: folic acid;C:child, PL:placebo,Zn:zinc<br>Intervention duration:to age 36 months
**Outcomes**	General intelligence<br>Assessment tool: UNIT<br>executive funtioning<br>Assessment tool: Stroop test, backward digit span, go/no-go tasks<br>motor (fine and gross) functioning<br>Assessment tool: MABC and finger tapping test<br>Assessment time: 7-9 years of age
**Title**	Preschool Iron-Folic Acid and Zinc Supplementation in Children Exposed to Iron-Folic Acid in Utero Confers No Added Cognitive Benefit in Early School-Age
**Results**	"The combination of M-IFAZn and C-IFA or C-IFAZn did not lead to any outcome differences relative to M-IFA alone.<br>Preschool iron-folic acid ±zinc to children exposed to iron-folic acid in utero or addition of zinc to maternal iron-folic acid conferred no additional benefit to cognitive outcomes assessed in early school age."
**Correspondence address**	pchristi@jhsph.edu
**Notes**	M:mother, C:child I:Iron, FA:folic acid, Zn:zinc, PL:placebo<br>"2 micronutrient supplementation trials indirectly in utero via antenatal supplementation and as 12- to 36-mo-old preschoolers were prospectively followed from June 2007 to April 2009 for assessment of developmental outcomes."<br>"Exposure to in utero supplementation was part of a maternal doublemasked, cluster-randomized, controlled, antenatal supplementation trial conducted from 1999 to 2001 in which pregnant women received daily folic acid (400 mg), folic acid-iron (60 mg), folic acid-iron-zinc (30 mg), or folic acid-iron-zinc and

	cholecalciferol (10 mg), tocopherol (10 mg),<br>thiamine (1.6 mg), riboflavin (1.8 mg), niacin (20 mg), pyridoxine (2.2 mg), vitamin B-12 (2.6 m<br>g), ascorbic acid (100 mg), phylloquinone (65 mg), copper (2.0 mg), and magnesium (100 mg), all with 1000 mg retinol<br>equivalents of vitamin A as retinyl palmitate compared to vitamin A alone as the control throughout pregnancy through 3 mo postpartum. Offspring of participating mothers from age 12 to 36 mo were then part of a larger 2*2 factorial, placebo-controlled, double-masked, cluster-randomized trial conducted from 2001 to 2005 in which they received daily iron-folic acid (12.5 mg), zinc (10 mg), iron-folic acid zinc, or placebo (29). All children received semiannual doses of vitamin A as prescribed by national policy."

Risk of bias table

Bias	Authors' judgement	Support for judgement
Random sequence generation (selection bias)	Unclear risk	Quote:"We randomised children by sector, stratified by geographic area and in blocks of four, to receive ..."
Allocation concealment (selection bias)	Low risk	Quote:"The sweet, vanilla flavoured supplements ... were packaged in blister packs of seven tablets. The foil backing on the blister pack was imprinted with the treatment code."<br>"Investigators, study staff, and participants were unaware of assigned treatments."
Blinding of participants and personnel (performance bias)	Low risk	Quote:"Investigators, study staff, and participants were unaware of assigned treatments."
Blinding of outcome assessment (detection bias)	Unclear risk	No relevant quote found in article
Incomplete outcome data (attrition bias)	Low risk	Attrition: 9.4%<br>No differential dropout was illustrated across supplementation groups in article figure
Selective reporting (reporting bias)	Low risk	All outcomes specified in the methods were reported
Other bias	Low risk	No evidence of other bias

Colombo 2014

**Methods**	Randomized Controlled Trial
**Participants**	Country:Peru, Setting: urban<br>Intervention date: N/A<br>Nutritional status:Inadequate for calcium , iron and zinc<br>Baseline length for age z score: - 0.5±0.9 in Iron+copper+ zinc group,- 0.6±0.8 in Iron+copper group<br>Inclusion criteria: 6 month old , birth weight >2500 g, gestational age > 37 week

	completed, free from major malformations, genetic abnormalities, or health problems associated with developmental delays, no known vision or hearing problems, hemoglobin concentration > 103 g/L, no plans to move<br>Exclusion criteria: none<br>Number randomized: 251 infants
**Interventions**	Intervention group :<br>iron + copper + zinc (10mg iron + 0.5 mg copper + 10 mg zinc)<br>number randomized:129; number analyzed:101<br>Control group :<br>iron + copper (10 mg iron + 0.5 mg copper)<br>number randomized:122 ; number analyzed:108<br>Formulation:Liquid supplement, iron as ferrous sulfate, copper as copper oxide, zinc as zinc sulfate<br>Frequency of supplementation: Once daily<br>Intervention duration:12 month from 6 month of age
**Outcomes**	Development (motor, mental and cognition)<br>Assessment tool: The Bayley Scales of Infant Development, version II (Bayley II)<br>Assessment time: 6 ,12 and 18 months
**Title**	Zinc Supplementation Sustained Normative Neurodevelopment in a Randomized, Controlled Trial of Peruvian Infants Aged 6–18 Months
**Results**	"The 2 groups did not differ on any of the BSID2 tasks."
**Correspondence address**	Schiefelbusch Institute for Life Span Studies and Department of Psychology, University of Kansas, Lawrence, KS<br>E-mail: colombo@ku.edu
**Notes**	

Risk of bias table

Bias	Authors' judgement	Support for judgement
Random sequence generation (selection bias)	Unclear risk	Quote:"Randomization by blocks of 2 within strata based on sex"
Allocation concealment (selection bias)	Low risk	Quote: "ID number and supplement type was sealed in a document and kept with the manufacture and the director of the institute"
Blinding of participants and personnel (performance bias)	Low risk	Quote: "The supplement bottles and liquids themselves were indistinguishable."<br>"The investigators, the families of study participants and data analysts had no knowledge of treatment groups."
Blinding of outcome assessment (detection bias)	Low risk	Quote: "The investigators, the families of study participants and data analysts had no knowledge of treatment groups."

Incomplete outcome data (attrition bias)	Low risk	Attrition:17%<br>"The enrollment characteristics of those who completed the study were compared with those lost to follow up. There were no differences in child, family characteristics, or initial hemoglobin concentrations, but those lost to follow up were significantly more likely to be girls and to be allocated to the supplemental zinc group. There were more children who left the study due to lack of time or because they disliked the supplement in the zinc group. The majority of these cases in the zinc group were girls.":refer to article Caulfield LE et al. 2013
Selective reporting (reporting bias)	Low risk	All outcomes specified in the methods were reported
Other bias	Low risk	No evidence of other bias

Gardner 2005

**Methods**	Randomized Controlled Trial
**Participants**	Country: Jamaica, setting:city<br>Intervention date:N/A<br>Nutritional status:N/A<br>Baseline length for age z score:- 1.6±0.82 in Zinc and - 1.25±0.81 in placebo group<br>Inclusion criteria:aged 9−30 month,underweight,<br>Those with current weight-for-age z scores below<br>1.5 SDs of the National Center for Health Statistics references and who had weight-for-age below 2 SDs in the previous 3 mo were enrolled into the study<br>Exclusion criteria:Twins or children with physical or mental impairments that could affect development were excluded.<br>Number randomized:126 children
**Interventions**	Intervention group 1:<br>Zinc (10 mg elemental zinc)<br>number randomized:35 number analyzed:30<br>Intervention group 2:<br>Zinc + stimulation (10 mg elemental zinc + stimulation)<br>number randomized:26 number analyzed:25<br>Intervention group 3:<br>placebo + stimulation<br>number randomized:23 number analyzed:21<br>Control group:<br>placebo<br>number randomized:42 number analyzed:38<br>Formulation:syrup, zinc sulfate<br>Frequency of supplementation:daily<br>Intervention duration:6 month

**Outcomes**	Development<br>Assessment tool: 4 subscales of the Griffiths Mental Development Scales<br>Assessment time:At enrollment and 6 month
**Title**	Zinc supplementation and psychosocial stimulation: effects on the development of undernourished Jamaican children
**Results**	"All children who received zinc benefited in hand and eye coordination."
**Correspondence address**	JM Meeks Gardner, Caribbean Child Development Centre, School of Continuing Studies, University of the West Indies,<br>Mona, Kingston 7, Jamaica. E-mail:julie.meeksgardner@uwimona.edu.jm.
**Notes**	"All children received a proprietary brand of micronutrients containing iron and vitamins, and caregivers were instructed to give them 0.5 mL daily."(8 mg iron in each ml)<br>"Ninety-nine children were identified<br>and enrolled over a period of 5 mo. For logistic reasons, we could not extend the stimulation program. To achieve sufficient power to detect an effect of zinc, we continued enrolling children for a further 2 mo to the zinc trial only."

Risk of bias table

Bias	Authors' judgement	Support for judgement
Random sequence generation (selection bias)	Unclear risk	No relevant quote found in article
Allocation concealment (selection bias)	Unclear risk	No relevant quote found in article
Blinding of participants and personnel (performance bias)	Low risk	Quote: "Informed consent was obtained from parents or guardians, who were unaware of the children's assignment to zinc or placebo."<br>"Zinc supplementation consisted ... as<br>sulfate in a flavored syrup or a placebo (syrup only) ..."
Blinding of outcome assessment (detection bias)	Low risk	Quote:"Developmental levels by 4 subscales of the Griffiths Mental Development Scales by a single tester, who was unaware of the children's group assignment."
Incomplete outcome data (attrition bias)	Low risk	Attrition:9.5%<br>Quote:"The children who withdrew were not significantly different from those who completed the study in any of the enrollment characteristics shown in Table 2 or in baseline developmental quotients, weights, or lengths."
Selective reporting (reporting bias)	Low risk	All outcomes specified in the methods were reported
Other bias	Low risk	No evidence of other bias

Hamadani 2001

**Methods**	Randomized Controlled Trial
**Participants**	Country:Bangladesh , setting:city (slum area)<br>Intervention date:N/A<br>Nutritional status:N/A<br>Baseline length for age z score: - 1.1±0.9 in Zinc group,- 1.1±0.8 in placebo group<br>Inclusion criteria:less than 4 weeks of age<br>Exclusion criteria:none<br>Number randomized:301
**Interventions**	Intervention group:<br>zinc (5 mg zinc)<br>number analyzed at 7 month:104<br>Control group:<br>placebo (cellulose substance)<br>number analyzed at 7 month:109<br>Formulation:syrup, Zinc as zinc acetate<br>Frequency of supplementation:daily<br>Intervention duration:5 month from 4 weeks of age
**Outcomes**	Development (motor and mental)<br>Assessment tool: The Bayley Scales of Infant Development, version II (Bayley II)<br>Assessment time:7 and 13 month of age
**Title**	Randomized controlled trial of the effect of zinc supplementation on the mental development of Bangladeshi infants
**Results**	"There were no significant differences in any of the behavioral and developmental variables at 7 month between the groups; at 13 month, however, the mean MDI score was significantly higher in the placebo group than in the zinc group."
**Correspondence address**	SM Grantham-McGregor, Center for International Child Health, Institute of Child Health,30 Guilford Street, London WC1N 1EH, United Kingdom. E-mail: s.mcgregor@ich.ucl.ac.uk.
**Notes**	

Risk of bias table

**Bias**	**Authors' judgement**	**Support for judgement**
Random sequence generation (selection bias)	Unclear risk	Quote:"Three hundred one infants were enrolled at 4 wk of age and were randomly assigned to a treatment or placebo group"
Allocation concealment (selection bias)	Unclear risk	No relevant quote found in article
Blinding of participants and personnel (performance bias)	Low risk	Quote:"The placebo was a cellulose substance given in an identical syrup."

Blinding of outcome assessment (detection bias)	Low risk	Quote:"Two testers who were unaware of the children's group assignment carried out the tests at 7 mo of age"
Incomplete outcome data (attrition bias)	Low risk	Attrition:21.7% at 7 month and 26.83% at 13 month of age<br>Quote:"There were no significant differences<br>between the lost and the retrieved sample in any of the variables examined (parental education, assets, and weight and length at 1 mo of age)."<br>"The difference in sex approached significance at both 7 and 13 mo, with a higher proportion of boys lost to follow- up in the zinc group than in the placebo group (P < 0.1). We therefore controlled for sex in all analyses."
Selective reporting (reporting bias)	Low risk	All outcomes specified in the methods were reported
Other bias	Low risk	No evidence of other bias

Heinig 2006

**Methods**	Randomized Controlled Trial
**Participants**	Country: United States of America, setting:city<br>Intervention date:between November 1994 and August 1997<br>Nutritional status:breast fed infants,introduction of complementary foods at an average of 22 wk of age, consuming meat,poultry,or fish an average of 2–3 d/wk.<br>Baseline length for age z score:lengths were 64.2±2.4 for zinc group and 63.9±2.4 for placebo group at 4 month of age(between 0-1 z score)<br>Inclusion criteria: healthy term infant weighing> 2500 g at birth; healthy nonsmoking mother ≥ 19 y of age, with no chronic medical condition that could interfere with lactation;Mother planned to fully breastfeed for ≥ 10 mo (ie, would not give formula on a daily basis) and not to introduce complementary foods before 4 mo; and mother planned to remain in the study area throughout the study period.<br>Exclusion criteria: None<br>Number randomized:85 infants
**Interventions**	intervention group :<br>zinc (5mg elemental zinc)<br>number randomized: 41; number analyzed: 33<br>Control group:<br>placebo (equivalent dose)<br>number randomized:44 ; number analyzed:37<br>Formulation: drops, zinc as zinc sulfate<br>Frequency of supplementation: once daily<br>Intervention duration:182 days from 4 to 10 mo of age
**Outcomes**	Motor development<br>Assessment tool: the Alberta Infant Motor Scale (AIMS)<br>Assessment time: at 4 (baseline) and 10 months of age

**Title**	Zinc supplementation does not affect growth, morbidity, or motor development of US term breastfed infants at 4–10 mo of age
**Results**	"The differences between groups in gross motor development based on AIMS scores at each age were not significant. All scores were at or above the 25th percentile for age. None of the infants was classified as being at risk (<-1 SD) or abnormal (<2 SD)."
**Correspondence address**	MJ Heinig, Department of Nutrition, University of California, One Shields Avenue, Davis, CA 95616-8669. E-mail: mjheinig@ucdavis.edu.
**Notes**	

Risk of bias table

Bias	Authors' judgement	Support for judgement
Random sequence generation (selection bias)	Low risk	Quote: "Random assignment to groups was done by using the Moses Oakford algorithm, as described by Meinert and Tonascia"
Allocation concealment (selection bias)	Low risk	Quote: "an assistant, who was not in contact with the study subjects, labeled the bottles with 1 of 4 colors (2 colors were assigned to each group to reduce the chance that a group assignment would accidentally be revealed)"
Blinding of participants and personnel (performance bias)	Low risk	Quote: "Each mother-infant pair was assigned to a color group so that neither the primary investigator nor the mothers would know whether their infants received the zinc supplement."
Blinding of outcome assessment (detection bias)	Unclear risk	No relevant quote found in article.
Incomplete outcome data (attrition bias)	Low risk	Attrition:17.6%<br>Quote: "There were no significant differences in demographic characteristics or growth from age 4 to 10mo between the 70 infants who remained eligible and those who became ineligible after enrollment."
Selective reporting (reporting bias)	Low risk	All outcomes specified in the methods were reported
Other bias	Low risk	No evidence of other bias

Jiminez 2007

**Methods**	Randomized Controlled Trial
**Participants**	Country:Cuba, setting:City<br>Intervention date:N/A<br>Nutritional status:N/A<br>Inclusion criteria:low birth weight children

	Exclusion criteria:children with serious illnesses that would endanger his life; children that although they presented low birth weight, the mother was receiving zinc supplementation during pregnancy, and children with<br>Congenital malformations.<br>Number randomized:212
**Interventions**	Intervention group :<br>Zinc (10 mg zinc sulfate per day)<br>number randomized: N/A ; number analyzed:87<br>Control group:<br>placebo (10 ml physiological saline per day)<br>number randomized:N/A; number analyzed:76<br>Formulation:syrup, zinc as zinc sulfate<br>Frequency of supplementation:divided into 2 doses during the first 6 month of life<br>Intervention duration:from 30 days of life until 6 month of age
**Outcomes**	Development (motor and mental)<br>Assessment tool: The Bayley Scales of Infant Development, version II (Bayley II)<br>Assessment time: 1,3,6,9 and 12 months of age.
**Title**	Zinc effects on growth and development of infant with low birth weight
**Results**	"zinc supplementation had no significant effect on mental development however motor development is influenced by zinc supplementation."
**Correspondence address**	Medical University of Havana. 2nd grade specialist in Pediatrics. Head of the Unit of Enteral Nutrition, University Pediatric Hospital «Juan Manuel Márquez», Gastroenterology Service, Havana, Cuba.<br>e-mail: rjimgar@infomed.sld.cu
**Notes**	

Risk of bias table

Bias	Authors' judgement	Support for judgement
Random sequence generation (selection bias)	Low risk	Quote:"La aleatorización del estudio se hizo por bloques que se generaron en computadora."<br>translation: Randomization of the study was made by blocks that were generated in computer.
Allocation concealment (selection bias)	Low risk	Quote:"El esquema de aleatorización lo llevó a cabo la dirección de farmacia del dispensario que elabora la fórmula"<br>translation: The randomization scheme was carried out by the pharmacy address of the dispensary that produces the formula
Blinding of participants and personnel (performance bias)	Low risk	Quote:"antes del comienzo del estudio el investigador envió las copias de la aleatorización al personal designado para controlar y administrar el medicamento; los investigadores no tuvieron acceso a las mismas."<br>translation: Before the start of the study, the researcher sent the

		copies of the randomization to designated personnel for control and administer the medication; the researchers they did not have access to them.
Blinding of outcome assessment (detection bias)	Unclear risk	No relevant quote found in article
Incomplete outcome data (attrition bias)	Unclear risk	Attrition:23.11 %
Selective reporting (reporting bias)	Low risk	All outcomes specified in the methods were reported
Other bias	Low risk	No evidence of other bias

Katz 2010

**Methods**	Community-based Cluster Randomized Controlled Trial
**Participants**	Country: Nepal, setting: Rural<br>Intervention date: 2001 and 2006<br>nutritional status: N/A<br>Baseline length for age z score:N/A<br>Inclusion criteria: All children 1–35 month of age in October 2001 were eligible for enrollment. Infants born from this time until January 2006 were also eligible for the trial when they reached 1 month of age.All children were discharged from the trial at age 36 month.<br>Exclusion criteria: none<br>Number randomized: 2274 children randomized to zinc and placebo groups<br>1772 children randomized to zinc+iron+ folic acid, iron+folic acid and placebo groups
**Interventions**	Intervention group 1:<br>zinc+iron+ folic acid (10 mg zinc + 12.5 mg iron + 50 micro g folic acid)<br>number randomized:686; number treated:340; number analyzed:238<br>Intervention group 2:<br>iron+folic acid (12.5 mg iron +50 micro g folic acid)<br>number randomized:501; number treated:242; number analyzed:195<br>Intervention group 3:<br>zinc (10 mg zinc)<br>number randomized:1079; number treated:759; number analyzed:539<br>Infants received half of these doses<br>Control group zinc+iron+ folic acid, iron+folic acid and placebo randomization:<br>placebo<br>number randomized:585; number treated:269; number analyzed:216<br>Control group zinc and placebo randomization:<br>placebo<br>number randomized:1195; number treated:847; number analyzed:588<br>Formulation:dispersible tablets, Zinc as zinc sulfate, Iron as ferrous sulfate<br>Frequency of supplementation: daily

	Intervention duration: until 36 month of age
**Outcomes**	Age at first walking unassisted (motor development)<br>assessment tool: pictures of 14 sequential motor milestones<br>assessment time: Weekly interview with the child’s caregiver
**Title**	Daily Supplementation with Iron Plus Folic Acid, Zinc, and Their Combination Is Not Associated with Younger Age at First Walking Unassisted in Malnourished Preschool Children from a Deficient Population in Rural Nepal
**Results**	"Mean age at first walking unassisted did not differ among groups and was 444± 81 d (mean ± SD) in the placebo group, 444 ±81 d in the zinc group, 464 ± 85 d in the iron+folic acid group, and 446 ± 87 d in the iron+folic acid+zinc group"
**Correspondence address**	Department of International Health , Bloomberg School of Public Health, Johns Hopkins University,Baltimore, MD 21205<br>E-mail: jkatz@jhsph.edu.
**Notes**	"The Data Safety and Monitoring Board recommended stopping supplementation with the iron+folic acid-containing treatments in November, 2003 due to no survival effect. Children who reconsented and switched from an iron-containing supplement to either zinc or placebo were not included<br>in this analysis.Children who aged into the study after November 2003<br>received only placebo or zinc supplementation and were included in the<br>placebo vs. zinc comparison."<br>"The analysis was intent to treat. Because children first enrolling in the<br>study could be 1−35 mo of age, the analysis was confined to those who<br>had not yet started walking unassisted."

Risk of bias table

Bias	Authors' judgement	Support for judgement
Random sequence generation (selection bias)	Unclear risk	Quote: "Children received 1 of 4 daily supplements, depending on sector of residence"
Allocation concealment (selection bias)	Unclear risk	No relevant quote found in article
Blinding of participants and personnel (performance bias)	Low risk	Quote: “A treatment code was imprinted on the package, but all tablets looked identical and study staff were unaware of the intervention.”
Blinding of outcome assessment (detection bias)	Low risk	Quote: "study staff were unaware of the intervention"
Incomplete outcome data (attrition bias)	High risk	Attrition zinc+iron+ folic acid, iron+folic acid and placebo randomization:31.12%<br>attrition zinc and placebo randomization:29.8%<br>distribution in the groups are provided

Selective reporting (reporting bias)	Low risk	All outcomes specified in the methods were reported
Other bias	Low risk	No evidence of other bias

Lind 2004

**Methods**	Randomized Controlled Trial
**Participants**	Country:Indonesia , setting:rural<br>Intervention date:July 1997 to May 1999<br>Nutritional status:the diet is plant-based and contains little animal protein and low amounts of iron and zinc with low bioavailability.<br>Baseline length for age z score:- 0.28±0.81 in Iron,- 0.33±0.84 in Zinc,- 0.36±0.83 in Iron+ zinc group and - 0.41±0.96 in placebo group<br>Inclusion criteria: Healthy singleton infants<br>Exclusion criteria:metabolic or neurologic disorders; handicaps affecting development, feeding, or activity; or severe or protracted illness, as well as infants with hemoglobin < 90 g/L<br>Number randomized:680 infants
**Interventions**	Intervention group 1:<br>Iron (10 mg iron)<br>number randomized:170; number analyzed:163<br>Intervention group 2:<br>Zinc (10 mg zinc)<br>number randomized:170; number analyzed:162<br>Intervention group 3:<br>Iron+ zinc (10 mg iron and 10 mg zinc)<br>number randomized:170;number analyzed:161<br>Control group:<br>placebo<br>number randomized:170; number analyzed:164<br>Formulation:sweet-tasting syrup,iron as ferrous sulfate , zinc as zinc sulfate<br>Frequency of supplementation:Once daily<br>Intervention duration: 180 days of supplementation from 6 to 12 month of age
**Outcomes**	Development (motor, mental, and behavior)<br>Assessment tool: The Bayley Scales of Infant Development, version II (Bayley II)<br>Assessment time:6 and 12 month of age
**Title**	A community-based randomized controlled trial of iron and zinc supplementation in Indonesian infants: effects on growth and development
**Results**	"zinc had no significant effect on MDI or PDI."
**Correspondence address**	T Lind, Department of Public Health and Clinical Medicine, Epidemiology and Public Health Sciences, Umeå University, SE-901 87 Umeå, Sweden. E-mail: torbjorn.lind@epiph.umu.se.
**Notes**	

Risk of bias table

Bias	Authors' judgement	Support for judgement
Random sequence generation (selection bias)	Low risk	Quote: "Randomization was planned and generated by an independent statistician and was performed in blocks of 20."
Allocation concealment (selection bias)	Low risk	Quote: "The pharmaceutical company marked the 4 different supplements with letter codes to which the researchers and participants were blinded Researchers and field staff were blinded to the information on group assignment, because this information was kept in safes at<br>the administrative offices of Gadjah Mada andUmeå universities until after the intent-to-treat analysis."
Blinding of participants and personnel (performance bias)	Low risk	Quote: "The pharmaceutical company marked the 4 different supplements with letter codes to which the researchers and participants were blinded"
Blinding of outcome assessment (detection bias)	Low risk	Quote: "Researchers and field staff were blinded to the information on group assignment, because this information was kept in safes at the administrative offices of Gadjah Mada andUmeå universities until after the intent-to-treat analysis."
Incomplete outcome data (attrition bias)	Low risk	Attrition: 3.7 %<br>Quote:"There were no significant differences ... between the group that completed the trial and the groups that did not"
Selective reporting (reporting bias)	Low risk	All outcomes specified in the methods were reported
Other bias	Low risk	No evidence of other bias

Locks 2016

**Methods**	Randomized Controlled Trial (substudy)
**Participants**	Country: Tanzania, setting: city<br>Intervention date: 2007<br>Nutritional status:One-fifth of households spent less than 1000 Tanzanian shillings (approximately $US0.75 at the time of the study) on food per day.<br>Baseline length for age z score: -0.43± 1.23 for zinc groups and -0.25±1.16 for not zinc groups<br>Inclusion criteria: singleton, live-birth infants born to HIV-negative mothers<br>Exclusion criteria: Infants of multiple births and infants with congenital abnormalities or other severe medical conditions<br>Number randomized:2400 infants, 247 of them underwent developmental assessment

**Interventions**	intervention group 1 :<br>Zinc (each capsule contained 5 mg zinc sulfate)<br>number analyzed:62<br>Intervention group 2 :<br>multivitamins (each capsule contained 60mg of vitamin C, 8mg of vitamin E, 0.5mg of vitamin B1, 0.6mg of vitamin B2, 4mg of niacin, 0.6mg of B6, 130 µg of folic acid and 1 µg of vitamin B12.)<br>number analyzed:60<br>Intervention group 3 :<br>zinc + multivitamins<br>number analyzed:59<br>Control group:<br>placebo<br>number analyzed:66<br>Formulation:opaque capsule containing an orange-flavoured powder<br>Frequency of supplementation:From the time of randomization until 6months of age, infants received one capsule per day. From 7months of age until the end of follow-up, infants received two capsules per day.<br>Intervention duration:from ages 6 weeks to 18 months
**Outcomes**	Development (cognition, language, and motor)<br>Assessment tool: The Bayley Scales of Infant Development, version 3 (BSID-III)<br>Assessment time: 15 month of age
**Title**	The effect of daily zinc and/or multivitamin supplements on early childhood development in Tanzania: results from a randomized controlled trial
**Results**	"Comparing children who received zinc supplements versus those who did not, no significant difference were detected in any of the five domains of BSID-III."
**Correspondence address**	ChristopherP. Duggan,Center for Nutrition,Division of Gastroenterology, Hepatology and Nutrition,Boston Children<br>'s Hospital, 333 Longwood Ave., Boston, MA 02115, USA; E-mail: christopher.duggan@childrens.harvard.edu
**Notes**	"At approximately 15 months of age, a sub-sample of 247 children was selected from a single research site (Magomeni Hospital)due to training and space restrictions."

Risk of bias table

Bias	Authors' judgement	Support for judgement
Random sequence generation (selection bias)	High risk	Quote: "At approximately 15 months of age, a sub-sample of 247 children was selected from a single research site ..."
Allocation concealment (selection bias)	Low risk	Quote:<br>"The biostatistician in Boston prepared a randomization list from 1 to 2400 that used blocks of 20 and was stratified by study clinic. Capsules were packaged in a blister pack of 15

		each and numbered boxes containing 6 blister packs were prepared containing the corresponding treatments. Each eligible infant was assigned the next numbered box of capsules at his/her respective site. The supplement used was an orange-flavored powder encapsulated in an opaque gelatinous capsule and was manufactured by Nutriset."
Blinding of participants and personnel (performance bias)	Low risk	Quote: "All four regimens were tested to ensure they were indistinguishable in appearance, smell and taste."<br>"All investigators and participants were blinded to treatment group"
Blinding of outcome assessment (detection bias)	Low risk	Quote:"All investigators and participants were blinded to treatment group"
Incomplete outcome data (attrition bias)	Low risk	Quote: "In our additional analyses, we did not find any significant differences between the sub-sample selected for BSID-III assessment and those excluded from the sub-sample in any of the baseline characteristics with the exception of the amount of Tanzanian Shillings spent on food per day (20.9% of infants in the sub-study came from families spending less than 1000 TSh per day compared with 29.5% among those who were not selected for the sub-study, P= 0.006)"
Selective reporting (reporting bias)	Low risk	All outcomes specified in the methods were reported
Other bias	Low risk	No evidence of other bias

Mathur 2015

**Methods**	Randomized Controlled Trial
**Participants**	country:India , setting:city<br>Intervention date: March 2011 through December 2011<br>Nutritional status: Exclusively breastfed during study period<br>Baseline length for age z score:N/A<br>Inclusion criteria: Preterm neonates, Less than 7 days old<br>Exclusion criteria: major congenital malformations/ not receiving anything orally for 7 days<br>Number randomized:100 pretem neonates, gestational age 33.4 ± 2.2 weeks for zinc goup and 33.4±2.3 weeks for control group.
**Interventions**	Intervention group:<br>Zinc (2 mg/kg/ day)<br>number randomized:50; number analyzed:37<br>Control group:<br>no placebo<br>number randomized:50; number analyzed:35

	Formulation: syrup, zinc as Zinc gluconate<br>Frequency of supplementation: once daily<br>Intervention duration: untill 3 month of corrected age
**Outcomes**	Neurodevelopment<br>Assessment tool: Amiel-Tison method by a single observer<br>Assessment time: at 40 weeks post conceptional age and at 3 months corrected age
**Title**	Zinc Supplementation in Preterm Neonates and Neurological Development: A Randomized Controlled Trial
**Results**	"At 40 weeks post conceptional age, none of the infants in Zinc group and 13% in control group showed moderate deficit in attention span (P= 0.02).<br>Higher number of neonates in control group had signs of hyperexcitability compatible with normal life at 40 week post-conceptional age (P =0.001)and 3 months corrected age (P =0.003).<br>Higher number of neonates in control group had signs of brisk bicipital reflex response at 40 weeks post conceptional age (P =0.01) and at 3 month corrected age(P =0.01).<br>Higher number of neonates in control group had signs of Brisk patellar reflex at 40 week post-conceptional age (P =0.002)and 3 months corrected age (P =0.006).<br>There was no difference in both groups at 40 weeks post conceptional age and 3 month corrected age with respect to visual and ocular signs, hearing abnormality, muscle tone, motor activity, involuntary movements, dystonia, cutaneous reflex, primitive reflex and asymmetric tonic neck reflex."
**Correspondence address**	Department of Neonatology, Maulana Azad Medical College, New Delhi 110 002, India.<br>E-mail: drnbmathur@gmail.com
**Notes**	Infants in both groups were given an oral calcium and vitamin D preparation, multivitamin drops (containing: Vitamin B1 2 mg,Niacinamide 10 mg, D-pantheno,and vitamin E drops.<br>Iron drops 2 mg/kg body weight daily was started at 4 weeks of postnatal age.

Risk of bias table

Bias	Authors' judgement	Support for judgement
Random sequence generation (selection bias)	Low risk	Quote: "Website generated random allocation sequence"
Allocation concealment (selection bias)	Low risk	Quote: "Concealed sealed opaque envelope"
Blinding of participants and personnel (performance bias)	High risk	Quote:"Eligible neonates ...were randomized either to receive zinc gluconate (2 mg/kg/day of elemental zinc) or no zinc ... ."<br>"We did not use any placebo in control group. The study was not blinded"

Blinding of outcome assessment (detection bias)	High risk	Quote:"The examiner was not blinded."
Incomplete outcome data (attrition bias)	Low risk	Attrition:28%<br>Loss to follow up are balanced in two groups.
Selective reporting (reporting bias)	Low risk	Cranial ultrasound and Brainstem evoked auditory response (BERA) are not reported however they are not related to developmental outcomes.
Other bias	Low risk	No evidence of other bias

Murray-Kolb 2012

**Methods**	Cluster Randomized Controlled Trial(follow up)
**Participants**	Country:Nepal , Setting: rural<br>Intervention date: (12 to 35 month old)<br>Assessment date: 2007 (follow up at 7-9 year old children)<br>Nutritional status:N/A<br>Baseline length for age z score: - 1.93±0.87 in Iron,folic acid and Zinc ,- 1.85±0.91 in Iron and folic acid group and - 1.97±0.84 in zinc group and - 1.89±0.90 in placebo group<br>Inclusion criteria:only children whose mothers were in the placebo arm of the in utero supplementation trial were eligible for this present analysis<br>Exclusion criteria:none
**Interventions**	Intervention group 1:<br>iron+ folic acid(12.5 mg of iron and 50 µg of folic acid)<br>number enrollment:178 ; number analyzed:171<br>Intervention group 2:<br>zinc (10 mg)<br>number enrollment:160; number analyzed:146<br>Intervention group 3:<br>iron plus folic acid and zinc<br>number enrollment: 209; number analyzed:200<br>Control group :<br>placebo<br>number enrollment:188 ; number analyzed:177<br>(iron plus folic acid and zinc versus iron+ folic acid;<br>Zinc versus placebo)<br>Formulation:tablet<br>Frequency of supplementation: daily<br>Intervention duration:12 to 35 months of age
**Outcomes**	General intelligence<br>Assessment tool: UNIT(Universal Nonverbal Intelligence Test)<br>executive funtioning<br>Assessment tool: Stroop (numbers) test, backward digit span, go/no-go tasks

	motor (fine and gross) skills<br>Assessment tool: MABC and finger tapping test<br>Assessment time: 7-9 years of age
**Title**	Preschool Micronutrient Supplementation Effects on Intellectual and Motor Function in School-aged Nepalese Children
**Results**	"study found no beneficial effect of either iron plus folic acid or zinc supplementation among children from 12 to 35 months of age on intellectual, executive, or motor function at 7 to 9 years of age."
**Correspondence address**	Parul Christian, DrPH, Center for Human Nutrition, Johns Hopkins Bloomberg School of Public Health, 615 N Wolfe St, Room W2041, Baltimore, MD 21205<br>E-mail:pchristi@jhsph.edu
**Notes**	"As part of the national program, children received a large dose of vitamin A (200 000 IU) once every 6 months throughout the study."<br>"Children in the present study represent a subset of children (n=3675) whose mothers participated in a micronutrient supplementation trial during pregnancy. Eligibility for the present analysis included children whose mothers were in the control arm of that trial"

Risk of bias table

**Bias**	**Authors' judgement**	**Support for judgement**
Random sequence generation (selection bias)	Unclear risk	Quote: "We randomised children by sector, stratified by geographic area and in blocks of four, to receive ..."
Allocation concealment (selection bias)	Low risk	Quote:"The sweet, vanilla flavoured supplements ... were packaged in blister packs of seven tablets. The foil backing on the blister pack was imprinted with the treatment code."<br>"Investigators, study staff, and participants were unaware of assigned treatments."
Blinding of participants and personnel (performance bias)	Low risk	Quote:"Investigators, study staff, and participants were unaware of assigned treatments."
Blinding of outcome assessment (detection bias)	Unclear risk	No relevant quote found in article
Incomplete outcome data (attrition bias)	Low risk	Attrition bias:10%<br>No differential dropout was illustrated across supplementation groups in article figure
Selective reporting (reporting bias)	Low risk	All outcomes specified in the methods were reported
Other bias	Low risk	No evidence of other bias

Olney 2006

**Methods**	Community-based Randomized Controlled Trial (substudy)
**Participants**	Country: Tanzania (Zanzibar,Pemba), setting:Urban and rural<br>Intervention date:2002 to 2003<br>Nutritional status:poor<br>Baseline length for age z score:- 1.5±1.0 in Iron+folic acid ,- 1.3±1.0 in zinc group,- 1.4±1.2 in Iron+folic acid+zinc group and - 1.6±1.0 in placebo group<br>Inclusion criteria: children aged 5–18 month at enrollment,<br>living in the included neighborhoods, and whose parents agreed to their participation in the main trial.<br>Exclusion criteria:none<br>Number randomized:876 (471 children were >11 month of age at baseline and were excluded from the analyses)
**Interventions**	Intervention group 1 :<br>iron + folic acid (6.25 mg Fe + 25 microgram folic acid)<br>number randomized:223; number analyzed:89<br>Intervention group 2:<br>Zinc (5 mg zinc)<br>number randomized:218; number analyzed:72<br>Intervention group 3:<br>iron + folic acid + zinc(6.25 mg Fe + 25 microgram folic acid + 5 mg zinc)<br>number randomized:220; number analyzed:90<br>Control group:<br>placebo<br>number randomized:215; number analyzed:103<br>Formulation:dispersible tablet<br>Frequency of supplementation:daily<br>Intervention duration:one year
**Outcomes**	Motor development (the time it took for children to walk unassisted)<br>assessment tool: picture chart containing 14 gross motor milestones<br>assessment time: every 2 weeks for 1 year or until they attained the highest milestone on the chart (standing on one foot), whichever came first.
**Title**	Combined Iron and Folic Acid Supplementation with or without Zinc Reduces Time to Walking Unassisted among Zanzibari Infants 5- to 11-mo old
**Results**	"Children who received any zinc didn't walk unassisted sooner than those who received no zinc."
**Correspondence address**	E-mail: rjs62@cornell.edu
**Notes**	"The present research was part of the Child Development<br>Substudy of a larger trial in Pemba designed to determine the effects of<br>FeFA, Zn, or FeFA1Zn on morbidity and mortality among children aged<br>1–35 mo "<br>"All children also received vitamin A: those aged 12 months or older were given 200 000 IU of vitamin A every 6 months and those aged younger than 12 months

	were given 100 000 IU."

Risk of bias table

Bias	Authors' judgement	Support for judgement
Random sequence generation (selection bias)	Unclear risk	Quote:"Randomisation was by household. We used a permuted block allocation sequence with a block length of 16 that was generated by WHO."
Allocation concealment (selection bias)	Low risk	Quote:"The supplement code, which was not known to the investigators, was maintained at WHO.To ensure masking, we labelled the strips of supplements with 16 letter codes—four for each of the groups. This letter code was hidden in the batch number on each strip of tablets.On enrolment, we assigned every child a code. Labels with the child’s name on were then printed from a computer database and attached by the pharmacy to the appropriate strip of supplements."
Blinding of participants and personnel (performance bias)	Low risk	Quote:"The supplement code, which was not known to the investigators, was maintained at WHO."
Blinding of outcome assessment (detection bias)	Low risk	Quote:"The supplement code, which was not known to the investigators, was maintained at WHO."
Incomplete outcome data (attrition bias)	Low risk	Attrition bias; 12.6 % (based on the number of less than 12 month old children)<br>No differential dropout was illustrated across supplementation groups in article figure
Selective reporting (reporting bias)	Low risk	All outcomes specified in the methods were reported
Other bias	Low risk	No evidence of other bias

Olney 2013

**Methods**	Community-based Randomized Controlled Trial (substudy)
**Participants**	Country: Tanzania (Zanzibar,Pemba), setting:Urban and rural<br>Intervention date:2002 to 2003<br>Nutritional status:poor<br>Baseline length for age z score: N/A ("The prevalence of stunting among the children aged 5–9 mo was 26% and was 36% among children aged 10–14 mo at baseline.")<br>Inclusion criteria:children aged 5–18 month at enrollment<br>Exclusion criteria:none<br>Number randomized:932 (children 15–19 mo at baseline–the majority of whom were walking at baseline–were excluded from this analysis(n = 241).)

**Interventions**	Intervention group :<br>iron + folic acid (12.5 mg Fe + 50 microgram folic acid)<br>number randomized:N/A; number analyzed:N/A<br>Intervention group :<br>Zinc (10 mg zinc)<br>number randomized:N/A; number analyzed:N/A<br>Intervention group :<br>iron + folic acid + zinc(12.5 mg Fe + 50 microgram folic acid + 10 mg zinc)<br>number randomized:N/A; number analyzed:N/A<br>Control group:<br>placebo<br>number randomized:N/A; number analyzed:N/A<br>Formulation:dispersible tablet<br>Frequency of supplementation:daily<br>Intervention duration:one year<br>half of the tablet were administered for less than 12 month olds.
**Outcomes**	Motor development (the time it took for children to walk unassisted)<br>Assessment tool: picture chart containing 14 gross motor milestones<br>Assessment time: every 2 weeks for 1 year or until the highest milestone was reached.
**Title**	Developmental effects of micronutrient supplementation and malaria<br>in Zanzibari children
**Results**	"FeFA with or without Zn had positive effects on motor development. Zn alone had negative effects on language development in children aged 10–14 mo."
**Correspondence address**	Poverty, Health and Nutrition Division, International Food<br>Policy Research Institute (IFPRI), 2033 K Street NW, Washington, DC 20006, United<br>States. Tel.: +1 202 862 5631; fax: +1 202 467 4439.<br>E-mail address: d.olney@cgiar.org (D.K. Olney).
**Notes**	"The present research was part of the Child Development<br>Substudy of a larger trial in Pemba designed to determine the effects of<br>FeFA, Zn, or FeFA1Zn on morbidity and mortality among children aged<br>1–35 mo "<br>"All children also received vitamin A: those aged 12 months or older were given 200 000 IU of vitamin A every 6 months and those aged younger than 12 months were given 100 000 IU."

Risk of bias table

Bias	Authors' judgement	Support for judgement
Random sequence generation (selection bias)	Unclear risk	Quote:"Randomisation was by household. We used a permuted block allocation sequence with a block length of 16 that was generated by WHO."

Allocation concealment (selection bias)	Low risk	Quote:"The supplement code, which was not known to the investigators, was maintained at WHO.To ensure masking, we labelled the strips of supplements with 16 letter codes—four for each of the groups. This letter code was hidden in the batch number on each strip of tablets.On enrolment, we assigned every child a code. Labels with the child's name on were then printed from a computer database and attached by the pharmacy to the appropriate strip of supplements."
Blinding of participants and personnel (performance bias)	Low risk	Quote:"The supplement code, which was not known to the investigators, was maintained at WHO."
Blinding of outcome assessment (detection bias)	Low risk	Quote:"The supplement code, which was not known to the investigators, was maintained at WHO."
Incomplete outcome data (attrition bias)	Low risk	On account of selection method of participants, no attrition bias was found.
Selective reporting (reporting bias)	Low risk	All outcomes specified in the methods were reported
Other bias	Low risk	No evidence of other bias

Pongcharoen 2011

**Methods**	Randomized Controlled Trial (follow up)
**Participants**	Country: Thailand, setting:rural<br>Intervention date:a follow-up cross-sectional study was conducted from August 2007 to January 2008 when the children were 9 years of age.<br>the original study was conducted from 1998 to 1999.<br>Nutritional status in original article:breast fed<br>Baseline length for age z score in original article:- 0.9±0.9 in zinc, - 0.9±0.9 in Iron,- 0.8±0.9 in Iron+ zinc group and - 0.8±0.9 in placebo group<br>Inclusion criteria in original study:d 4–6 month infants; predominantly breast fed, free from apparent congenital abnormalities<br>Exclusion criteria in original study:hemoglobin (Hb) <80 g/L, chronic illnesses, bottle fed<br>Exclusion criteria in follow up study: neurological disorder<br>number randomized in original study: 675<br>number completed the original study:609<br>number followed:560
**Interventions**	Intervention group1:<br>zinc (10 mg zinc)<br>number completed the original study:151; number assessed at follow up:139<br>Intervention group2:<br>Iron (10 mg ferrous sulfate)<br>number completed the original study:153; number assessed at follow up:147<br>Intervention group3:<br>zinc + Iron (10 mg zinc + 10 mg ferrous sulfate)

	number completed the original study:152; number assessed at follow up:135<br>Control group:<br>placebo<br>number completed the original study:153; number assessed at follow up:139<br>Formulation: syrup ; zinc as zinc sulfate , iron as ferrous sulfate<br>Frequency of supplementation:daily<br>Intervention duration:6 month
**Outcomes**	Cognitive performance<br>Assessment tool:Wechsler Intelligence Scale for Children−Third edition (WISC-III; Thai version) and Raven’s Colored Progressive Matrices (CPM)<br>Assessment time: 9 years old
**Title**	Long-term effects of iron and zinc supplementation during infancy on cognitive function at 9 y of age in northeast Thai children: a follow-up study
**Results**	"No significant differences in any of the outcomes at 9 y of age were observed at follow-up between the 4 groups."
**Correspondence address**	R Martorell, Hubert Department of Global<br>Health, Rollins School of Public Health, Emory University, 1599 Clifton Road, Atlanta, GA 30322. E-mail: rmart77@emory.edu.
**Notes**	"A follow-up study was performed in 560 children aged 9 y<br>or 92% of those who had participated in a randomized controlled trial involving 4 groups who received daily iron, zinc, iron plus zinc, or a placebo at 4−6 month of age for 6 month."

Risk of bias table

**Bias**	**Authors' judgement**	**Support for judgement**
Random sequence generation (selection bias)	Low risk	Quote: "The study was a randomized 2 * 2 factorial, double-blind, placebo controlled trial ... "<br>"The randomization was done by a statistician who was not involved in the study."
Allocation concealment (selection bias)	Low risk	Quote:"The randomization was done by a statistician who was not involved in the study."<br>"The bottles of syrup were coded at the production site. The code allocation was kept at UNICEF, Jakarta, until the end of data analysis."
Blinding of participants and personnel (performance bias)	Low risk	Quote:"The code allocation was kept at UNICEF, Jakarta, until the end of data analysis."
Blinding of outcome assessment (detection bias)	Low risk	Quote:"The psychologists were unaware of the child’s intervention group."

Incomplete outcome data (attrition bias)	Low risk	Attrition bias:8%<br>Quote:"We compared nonparticipating (n = 49) and participating children in the follow-up and found no differences in the distribution of supplementation type (P = 0.122), sex (P = 0.579), birth weight (P = 0.511), age at recruitment into the trial (P = 0.940), and biochemical and anthropometric status at recruitment into the trial hemoglobin (P = 0.676), serum zinc (P = 0.818), weight<br>(P= 0.668), and height (P= 0.922)"
Selective reporting (reporting bias)	Low risk	All outcomes specified in the methods were reported
Other bias	Low risk	No evidence of other bias

Prado 2016

**Methods**	Cluster Randomized Controlled Trial (substudy)
**Participants**	Country: Burkina Faso, setting:rural<br>Intervention date:April 2010 to July 2012<br>Nutritional status:Young children in the area are affected by holoendemic malaria transmission and a high prevalence of stunting and underweight<br>Baseline length for age z score: -1.18±1.08 in LNS-Zn0, -1.31±1.12 in LNS-Zn10, -1.07± 1.09 in LNS-TabZn5, -1.22± 1.10 in NIC<br>Inclusion criteria:8.8 to 9.9 mo of age, resided permanently in the Dand´e Health District, planned to be available during the study period, and if written parental consent was obtained.<br>Exclusion criteria: hemoglobin <50 g/L (11), weight-for-length <70% of the median of the National Center for Health Statistics/WHO growth reference, presence of bipedal edema, severe illness warranting hospital referral, congenital abnormalities potentially interfering with growth, chronic medical condition (e.g., malignancy) requiring frequent medical attention, known HIV infection of the infant or mother, history of allergy to peanuts, history of anaphylaxis or serious allergic reaction to any substance, requiring emergency medical care, and concurrent participation in any other clinical trial. Once enrolled, children were excluded if absent from the study for >3 wk.<br>Number randomized:1426<br>8.8 to 9.9 month of age
**Interventions**	Intervention group 1:<br>LNS-Zn0 (20 g SQLNSs/d + 0 mg added zinc+ a placebo tablet)<br>number treated: 328; number analyzed: 255<br>Intervention group 2:<br>LNS-Zn10 (20 g SQ-LNSs/d +10 mg added zinc+ a placebo tablet)<br>number treated:326; number analyzed: 248<br>Intervention group3:<br>LNS-TabZn5 (20 g SQ-LNSs/d + 0 mg added zinc + a tablet containing 5 mg Zn)<br>number treated:326; number analyzed: 243

	Control group:<br>number treated: 446; number analyzed: 376<br>Formulation:zinc as zinc sulfate<br>Frequency of supplementation:daily<br>Intervention duration:9 month
**Outcomes**	Development(motor,language, and personal-social development)<br>Assessment tools:FCI score, Developmental Milestones Checklist (DMC) II<br>Assessment time: 18 months of age
**Title**	Lipid-Based Nutrient Supplements Plus Malaria and Diarrhea Treatment Increase Infant Development Scores in a Cluster-Randomized Trial in Burkina Faso
**Results**	"Children in the IC scored 0.34 (95%CI: 0.21, 0.46), 0.30 (95%CI: 0.15, 0.44), and 0.32 (95%CI: 0.16, 0.48) SDs higher in motor, language, and personal-social development, respectively, than did children in the NIC (All P < 0.001). Children who received different amounts of zinc did not differ significantly in any of the scores. No effect on caregiver-child interaction was found."
**Correspondence address**	Departments of Nutrition and Agricultural and Resource Economics, University of California Davis, Davis, CA
**Notes**	"The SQ-LNS products were developed and produced by Nutriset SAS. Twenty grams of SQ-LNSs contained 118 kcal, 2.6 g protein, 9.6 g fat, 4.46 g linoleic acid (18:2n26), 0.58 g a-linoleic acid (18:3n23), 400 mg vitamin A (retinyl acetate), 0.3 mg thiamine, 0.4 mg riboflavin, 4 mg niacin, 1.8 mg pantothenic acid, 0.3 mg vitamin B-6, 0.5 mg vitamin B-12, 80 mg folic acid (pteroyl monoglutamic acid), 30 mg vitamin C (L-ascorbic acid), 5 mg vitamin D (cholecalciferol), 6 mg vitamin E (d,la- tocopherol acetate), 30 mg vitamin K (phylloquinone 5%), 280 mg calcium (tricalcium phosphate), 0.34 mg copper, 90 mg iodine, 6 mg iron, 40 mg magnesium, 1.2 mg manganese, 190 mg phosphorus, 200 mg potassium, and 20 mg selenium."<br>"participants were instructed to feed the tablet to the child at least half an hour before or after any meal or snack, including SQ-LNSs."

Risk of bias table

Bias	Authors' judgement	Support for judgement
Random sequence generation (selection bias)	Low risk	Quote:"the trial included 2 levels of randomization: the community and the child. First, 34 communities ... by computer-generated random assignment within strata to ... Second, 2435 eligible children in the IC communities were assigned by a random allocation sequence to 1 of the following supplement groups ..."<br>"A subsample of 446 children from the NIC and 980 children from 3 of the 4 IC groups ... were randomly selected for motor, language, and personal-social assessment at 18 mo of age. This selection was accomplished through a specialized SAS

		program (SAS Institute), which randomly assigned children to be assessed or not assessed within treatment group and time block."
Allocation concealment (selection bias)	Unclear risk	No relevant quote found in article
Blinding of participants and personnel (performance bias)	High risk	Quote:"Data collectors and participants were aware of the allocation of IC or NIC but were blind to the IC groups."
Blinding of outcome assessment (detection bias)	Low risk	Quote:"The data collectors were unaware which groups had been selected for developmental assessment, thus maintaining blinding."
Incomplete outcome data (attrition bias)	Unclear risk	Attrition bias: 21.3%
Selective reporting (reporting bias)	Low risk	All outcomes specified in the methods were reported
Other bias	Low risk	No evidence of other bias

Sazawal 1996

**Methods**	Randomized Controlled Trial
**Participants**	Country: India(New Delhi) , setting:urban<br>Intervention date:1993<br>Nutritional status:low socioeconomic population<br>Baseline length for age z score: 52.1% stunted children in zinc group and 44.5% in control group<br>Inclusion criteria: children 6 to 35 months old with diarrhea presenting to the dispensary with reported passage of at least 4 unformed stools in the previous 24 hours, a diarrhoeal duration of less than 7 days and permanent residence in the trial area<br>Exclusion criteria:children with malnutrition sufficiently severe to require hospitalization<br>Number randomized: 93 (all children 12 to 23 months of age that recieved supplementation for at least 1 month were selected for activity assessments."
**Interventions**	Intervention group :<br>zinc (10 mg zinc however during diarrheal illness this was incresed to 20 mg)<br>number randomized:48; number analyzed:48<br>Control group:<br>placebo<br>number randomized:45; number analyzed:45<br>Formulation:syrup, zinc as zinc gluconate<br>Frequency of supplementation: daily<br>Intervention duration:1 to 6 months

**Outcomes**	Develoment<br>Assessment tool: percentage of time spent in each of 5 activity levels and 2 groups representing high and low movement and overall rating by 2 activity scores<br>Assessment time: 12 to 23 months of age
**Title**	Effect of zinc supplementation on observed activity in low socioeconomic Indian preschool children.
**Results**	"Children in the zinc group spent 72% more time performing activities in the high-movement group. Among the zinc-supplemented children, the activity rating by the children's activity rating score was 12% higher and by the energy expenditure score was 8.3% higher than in the control group."
**Correspondence address**	Sazawal S<br>Indian Council of Medical Research Advanced Center for Diarrheal Disease Research, Division of Pediatric Gastroenterology, All India Institute of Medical Sciences, New Delhi, India.
**Notes**	Both groups received vitamins A 800 U, B1 0.6 mg, B2 0.5 mg, B6 0.5 mg, D3 100 IU and E 3 mg and niacinamide 10 mg daily

Risk of bias table

**Bias**	**Authors' judgement**	**Support for judgement**
Random sequence generation (selection bias)	Unclear risk	Quote:“Randomization schedules with permuted blocks of fixed length appropriate for double-blind studies were used to compensate ...”<br>“Randomization was stratified by nutritional and breastfeeding status,...”
Allocation concealment (selection bias)	Low risk	Quote:“A sealed envelope contained the assigned group for each enrolled child"
Blinding of participants and personnel (performance bias)	Low risk	Quote: "the bottles labeled with identification number and name were given to the mother and kept at the child's home"<br>“A separate team of field assistants dedicated to dispensing the assigned preparation visited the family every day except Sundays and holidays and fed the preparation to the child”
Blinding of outcome assessment (detection bias)	Low risk	Quote: “Observers were unaware of group allocations"
Incomplete outcome data (attrition bias)	Low risk	On account of selection method of participants, no attrition bias was found.
Selective reporting (reporting bias)	Low risk	All outcomes specified in the methods were reported
Other bias	Low risk	No evidence of other bias

Siegel 2011

**Methods**	Cluster Randomized Controlled Trial
**Participants**	Country:Nepal , setting:rural<br>Intervention date: 2002<br>Nutritional status:poor<br>Baseline length for age z score:22% were stunted (less than -2 standard deviation)<br>Inclusion criteria: children aged 53 weeks or less at baseline<br>Exclusion criteria:none<br>Number randomized:259
**Interventions**	Intervention group1 :<br>iron+ folic acid (6.25 mg iron sulfate and 25 microgram folic acid)<br>number randomized:N/A; number analyzed:N/A<br>Intervention group 2<br>zinc (5 mg zinc sulfate)<br>number randomized:N/A; number analyzed:N/A<br>Intervention group 3:<br>iron+ folic acid + zinc (6.25 mg iron sulfate and 25 microgram folic acid + 5 mg zinc sulfate)<br>number randomized:N/A; number analyzed:N/A<br>Control group:<br>placebo<br>number randomized:N/A; number analyzed:N/A<br>Formulation: dispersible tablet, zinc as zinc sulfate, Iron as Iron sulfate<br>Frequency of supplementation:daily<br>Intervention duration:ranged from 0 to 24 weeks for children who completed the 39 weeks assessments and 0 to 37 weeks for children who completed the 52 weeks assessments
**Outcomes**	Cognitive development<br>Assessment tool: information-processing measures that were part of<br>the FTII and the A-not-B Task<br>Assessment time: at 39 and 52 week old
**Title**	Inconsistent Effects of Iron-Folic Acid and/or Zinc Supplementation on the Cognitive Development of Infants
**Results**	"Neither the combined nor the individual micronutrient supplements improved the performance on the FTII or the A-not-B Task (p>0.05)."
**Correspondence address**	Department of International Health<br>Room W5009 Johns Hopkins Bloomberg School of Public Health 615 North Wolfe Street, Baltimore, MD 21205<br>USA Email: SiegelEH@gmail.com
**Notes**	

Risk of bias table

Bias	Authors' judgement	Support for judgement
Random sequence generation (selection bias)	Unclear risk	Quote: "In the larger 2x2 factorial trial, the unit of randomization was a geographic sector, with several sectors comprising a VDC. Randomization was stratified by the VDC. Randomization for the 23 sectors within Ishwarpur VDC was carried out in 6 blocks of 4"
Allocation concealment (selection bias)	Low risk	Quote: "Neither the families of the participants nor the research team were able to trace the code assignments to the supplements."
Blinding of participants and personnel (performance bias)	Low risk	Quote: "Neither the families of the participants nor the research team were able to trace the code assignments to the supplements."
Blinding of outcome assessment (detection bias)	Low risk	Quote: "Neither the families of the participants nor the research team were able to trace the code assignments to the supplements."
Incomplete outcome data (attrition bias)	Low risk	Attrition bias:couldn't be calculated<br>Quote:"Descriptive characteristics, including sex, caste, SES, anaemia, IDA, and anthropometry did not differ (p>0.05) between children who completed and did not complete the tests (data not shown)."
Selective reporting (reporting bias)	Low risk	All outcomes specified in the methods were reported
Other bias	Low risk	No evidence of other bias

Sudfeld 2019

**Methods**	Randomized Controlled Trial (follow up)
**Participants**	Country: Tanzania, Setting: periurban<br>Intervention date: 2007 to 2011 (6 weeks to 18 month old)<br>Assessment date: July 2015 to March 2017 (follow up at 6-8 year old children)<br>Nutritional status: N/A<br>Inclusion criteria: HIV-unexposed infants<br>Exclusion criteria:Infants of multiple births and infants with congenital anomalies
**Interventions**	Intervention group 1:<br>Zinc + multivitamins<br>number enrollment:66 ; number analyzed:66<br>Intervention group 2:<br>Zinc<br>number enrollment:101; number analyzed:101

	Intervention group 3:<br>Multivitamins<br>number enrollment:106 ; number analyzed:106<br>Control group :<br>Placebo<br>number enrollment:92 ; number analyzed:92<br>Formulation:Capsules, zinc as N/A<br>Frequency of supplementation: one capsule daily (or two capsules if > 6 months)<br>Intervention duration:to age 18 months
**Outcomes**	General intelligence<br>Assessment tool:<br>Koh’s Block Design test<br>Verbal Fluency test<br>executive funtioning<br>Assessment tool: East African Neurodevelopment Tools<br>Assessment time: 6-8 years of age
**Title**	Effect of antenatal and infant micronutrient supplementation on middle childhood and early adolescent development outcomes in Tanzania
**Results**	We found no effect of infant zinc or MMN supplementation any development domain at 6–8<br>years of age
**Correspondence address**	csudfeld@hsph.harvard.edu
**Notes**	The infant micronutrient supplementation trial began<br>enrollment in July 2007 and completed follow-up for the primary outcomes in May 2011 [14]. The trial enrolled 2400 HIV-unexposed infants at 6 weeks of age and sup-plemented children to 18 months of age. Infants were ran-domized in a factorial design to receive a daily oral dose of one of four trial regimens: (1) zinc, (2) MMN, (3) zinc+MMN, or (4) placebo. Infants received one capsule per day from 6 weeks to 6 months of age and then two capsules per day from 7 months of age to the end of follow-up at 18 months post-randomization.

Risk of bias table

Bias	Authors' judgement	Support for judgement
Random sequence generation (selection bias)	Low risk	Quote:"Infants were randomly assigned in a<br>factorial design to receive a daily oral dose"
Allocation concealment (selection bias)	Low risk	Quote:"The biostatistician in Boston prepared a randomization list from 1 to 2400 that used blocks of 20 and was stratified by study clinic."
Blinding of participants and personnel (performance bias)	Low risk	Quote:"All study personnel and participants were blinded to treatment assignment for the duration of the study."

Blinding of outcome assessment (detection bias)	Low risk	Quote:"The study staff who administered the development assessment, the parents, and the children were blinded to their randomized trial group."
Incomplete outcome data (attrition bias)	Unclear risk	No relevant quote found in article
Selective reporting (reporting bias)	Low risk	All outcomes specified in the methods were reported
Other bias	Low risk	No evidence of other bias

Surkan 2013

**Methods**	Cluster Randomized Controlled Trial (substudy)
**Participants**	Country:Nepal , setting:rural<br>Intervention date: January 2002 and April 2003<br>Nutritional status:largely micronutrient deficient population<br>Baseline length for age z score:N/A<br>Inclusion criteria:4−17 months old<br>Exclusion criteria:N/A<br>Number randomized:569 children
**Interventions**	intervention group 1 :<br>zinc (10 mg zinc)<br>number randomized:127; number analyzed:124<br>Intervention group 2 :<br>iron-folic acid (12.5 mg iron and 50 µg folic acid)<br>number randomized:129; number analyzed:122<br>Intervention group 3 :<br>zinc plus iron-folic acid (10 mg zinc, 12.5 mg iron and 50 µg folic acid)<br>number randomized:161; number analyzed:152<br>Control group:<br>placebo (a sugar placebo)<br>number randomized: 152 ; number analyzed:146<br>Formulation:dispersible tablets, zinc as zinc sulfate<br>Frequency of supplementation:daily<br>Intervention duration:one year
**Outcomes**	Development (parental report of Motor and Language Milestones)<br>Assessment tools:Motor and language milestone instruments were adaptedfrom the Griffiths Mental Development Scale and the MacArthur Communicative Development Inventory<br>Assessment time: baseline and three month intervals for one year
**Title**	Zinc and iron supplementation on motor and language milestone scores of infants and toddlers
**Results**	"no evidence of effects of zinc supplementation on motor or language milestones."

**Correspondence address**	Pamela J Surkan, Social and Behavioral Interventions Program, Dept. of International Health, Johns Hopkins Bloomberg School of Public Health, 615 North Wolfe St., Room E5523, Baltimore, MD 21205-2179, 410-502-7396 (office), 410-502-6733 (fax), psurkan@jhsph.edu.
**Notes**	A sub-study of the Nepal Nutrition Intervention Project Sarlahi (NNIPS-4). Children under age 1 year received a half-dose of supplements.

Risk of bias table

Bias	Authors' judgement	Support for judgement
Random sequence generation (selection bias)	Low risk	Quote: "All possible combinations of the 4 treatment groups were written on pieces of paper. The sectors were geographically ordered and senior field personnel randomly and blindly withdrew pieces of paper from a container that indicated the codes for the first 4 sectors on the list. The paper was replaced and random drawing continued until all sectors had been assigned to one of the four treatment groups."
Allocation concealment (selection bias)	Unclear risk	No relevant quote found in article
Blinding of participants and personnel (performance bias)	Low risk	Quote:"Field staff and participating families were masked to the randomization of<br>the intervention. All tablets looked identical."
Blinding of outcome assessment (detection bias)	Low risk	Quote:"The study used a randomized and triple masked controlled design."
Incomplete outcome data (attrition bias)	Low risk	Attrition: 4.4%
Selective reporting (reporting bias)	Low risk	All outcomes specified in the methods were reported
Other bias	Low risk	No evidence of other bias

Taneja 2005

**Methods**	Randomized Controlled Trial
**Participants**	Country: india, setting:urban<br>Intervention date:15 February 1999<br>Nutritional status:Childhood malnutrition and zinc deficiency are common<br>baseline length for age z score:34.6% of zinc and 38.9% of placebo children had length for age z score less than -2 SD<br>Inclusion criteria:12 to 18 months of age<br>Exclusion criteria:planning to move within the<br>next 4 months, hospitalization on the enrollment day,receiving a massive dose of vitamin A within 2 months.

	Number randomized:2482 (650 selected for developmental assessments)
**Interventions**	Intervention group :<br>Zinc (10 mg zinc for infants and 20 mg for older children)<br>number randomized:327; number analyzed:283<br>Control group:<br>placebo<br>number randomized:323; number analyzed:288<br>Formulation:syrup, zinc as zinc gluconate<br>frequency of supplementation:daily<br>Intervention duration:4 months
**Outcomes**	Development (motor and mental)<br>Assessment tool: The Bayley Scales of Infant Development, version II (Bayley II)<br>Assessment time:baseline and 4 month after supplementation
**Title**	Impact of zinc supplementation on mental and psychomotor scores of children aged 12 to 18 months: a randomized, double-blind trial.
**Results**	"Zinc supplementation did not affect the mental or psychomotor development index scores."
**Correspondence address**	M. K. Bhan, Department of Pediatrics, All India Institute of Medical Sciences, New Delhi 110029, India. E-mail: community.research@cih.uib.no
**Notes**	

Risk of bias table

**Bias**	**Authors' judgement**	**Support for judgement**
Random sequence generation (selection bias)	Low risk	Quote:"simple randomization scheme in blocks of 8."<br>"The randomization scheme was generated by a statistician at the Statens Serum Institute, who was not otherwise involved with this study, using SAS software"
Allocation concealment (selection bias)	Low risk	Quote:"The randomization scheme was generated by a statistician at the Statens Serum Institute, who was not otherwise involved with this study, using SAS software"
Blinding of participants and personnel (performance bias)	Low risk	Quote: "Zinc or placebo syrups, similar in appearance and taste, were prepared and packaged in unbreakable bottles by GK Pharma Aps, Koge, Denmark; they also labeled bottles with unique child identification numbers according to the randomization scheme."
Blinding of outcome assessment (detection bias)	Unclear risk	No relevant quote found in article

Incomplete outcome data (attrition bias)	Unclear risk	Attrition bias:12.15%
Selective reporting (reporting bias)	Low risk	All outcomes specified in the methods were reported
Other bias	Low risk	No evidence of other bias

Footnotes
