# Supplementary material for: Does zinc with and without iron co-supplementation have effect on motor and mental development of children? A systematic review and meta-analysis
Source: BMC Pediatr. 2020 Sep 28;20:451. doi: 10.1186/s12887-020-02340-1 (PMC7520965; doi:10.1186/s12887-020-02340-1)
Supplement: Supplementary file 2 — Additional file 2: Table S1. MEDLINE (Ovid) search strategy. Table S2. GRADE assessments for zinc alone comparisons. Table S3. GRADE assessments for zinc with iron comparisons. Figure S1. Funnel plot of comparison Zinc alone versus Placebo, outcome: MDI at 6 months of age. Figure S2. Funnel plot of comparison Zinc alone versus Placebo, outcome: PDI at 6 months of age. Figure S3. Funnel plot of comparison Zinc alone versus Placebo, outcome: MDI at 12 months of age. Figure S4. Funnel plot of comparison Zinc alone versus Placebo, outcome: PDI at 12 months of age. Figure S5. Funnel plot of comparison Zinc with Iron versus Iron, outcome: MDI at 6 months of age. Figure S6. Funnel plot of comparison Zinc with Iron versus Iron, outcome: PDI at 6 months of age. Figure S7. Funnel plot of comparison: Zinc with Iron versus Iron, outcome: MDI at 12 months of age. Figure S8. Funnel plot of comparison: Zinc with Iron versus Iron, outcome: PDI at 12 months of age. [file 12887_2020_2340_MOESM2_ESM.docx]

**Supplementary Appendix**

**Table 1: MEDLINE (Ovid) search strategy**

| # | Searches |
| --- | --- |
| 1 | zinc.tw. |
| 2 | Zinc/ |
| 3 | 1 or 2 |
| 4 | Infant, Newborn/ |
| 5 | Infant/ |
| 6 | Child, Preschool/ |
| 7 | Child/ |
| 8 | (neonate* or infant* or newborn* or baby or babies or toddler* or boy* or girl* or child* or preschool* or pre-school* or school* or schoolchild* or preteen* or pre-teen* or preadolescen* or pre-adolescen*).tw. |
| 9 | 4 or 5 or 6 or 7 or 8 |
| 10 | randomized controlled trial.pt. |
| 11 | randomized controlled trial.ab. |
| 12 | controlled clinical trial.pt. |
| 13 | controlled clinical trial.ab. |
| 14 | randomized.ab. |
| 15 | randomly.ab. |
| 16 | placebo.ab. |
| 17 | trial.ab. |
| 18 | groups.ab. |
| 19 | drug therapy.fs. |
| 20 | 10 or 11 or 12 or 13 or 14 or 15 or 16 or 17 or 18 or 19 |
| 21 | 3 and 9 and 20 |

**Table 2: GRADE assessments for zinc alone comparisons**

**Question**: Zinc alone supplementation compared to Placebo for child mental and motor development

| **Certainty assessment** | | | | | | | **№ of patients** | | **Effect** | | **Certainty** | **Importance** |
| --- | --- | --- | --- | --- | --- | --- | --- | --- | --- | --- | --- | --- |
| **№ of studies** | **Study design** | **Risk of bias** | **Inconsistency** | **Indirectness** | **Imprecision** | **Other considerations** | **Zinc alone supplementation** | **Placebo** | **Relative (95% CI)** | **Absolute (95% CI)** |  |  |
| **MDI at 6 months of age (zinc alone versus placebo) (assessed with: Bayley (BSID-II) )** | | | | | | | | | | | | |
| 4 | randomised trials | serious ^a^ | not serious | not serious | not serious | none | 301 | 290 | - | SMD **0.18 SD lower** (0.39 lower to 0.02 higher) | ⨁⨁⨁◯ MODERATE |  |
| **PDI at 6 months of age (zinc alone versus placebo) (assessed with: Bayley (BSID-II))** | | | | | | | | | | | | |
| 4 | randomised trials | serious ^b^ | serious ^c^ | not serious | not serious | none | 301 | 290 | - | SMD **0.17 SD higher** (0.2 lower to 0.55 higher) | ⨁⨁◯◯ LOW |  |
| **MDI at 12 months of age (zinc alone versus placebo) (assessed with: Bayley (BSID-II))** | | | | | | | | | | | | |
| 6 | randomised trials | serious ^d^ | serious ^e^ | not serious | not serious | none | 497 | 480 | - | SMD **0.08 SD lower** (0.36 lower to 0.19 higher) | ⨁⨁◯◯ LOW |  |
| **PDI at 12 months of age (zinc alone versus placebo) (assessed with: Bayley (BSID-II))** | | | | | | | | | | | | |
| 6 | randomised trials | serious ^f^ | serious ^g^ | not serious | not serious | none | 497 | 480 | - | SMD **0.3 SD higher** (0.24 lower to 0.83 higher) | ⨁⨁◯◯ LOW |  |

**CI:** Confidence interval; **SMD:** Standardised mean difference

#### Explanations

a. three out of four studies have unclear risk of bias and one study has high risk of bias for child.

b. three out of four studies have unclear risk of bias and one study has high risk of bias.

c. The heterogeneity is high (I square is 80.8% and is above 75% threshold).

d. Two out of six studies have high risk of bias and three of them have unclear risk of bias.

e. The heterogeneity is high (I square is 77.4% and is above 75% threshold).

f. Two out of six studies have high risk of bias and three of them have unclear risk of bias.

g. The heterogeneity is high (I square is 92% and is above 75% threshold).

**Table 3: GRADE assessments for zinc with iron comparisons**

**Question**: Zinc with Iron supplementation compared to Iron for child mental and motor development

| **Certainty assessment** | | | | | | | **№ of patients** | | **Effect** | | **Certainty** | **Importance** |
| --- | --- | --- | --- | --- | --- | --- | --- | --- | --- | --- | --- | --- |
| **№ of studies** | **Study design** | **Risk of bias** | **Inconsistency** | **Indirectness** | **Imprecision** | **Other considerations** | **Zinc with Iron supplementation** | **Iron** | **Relative (95% CI)** | **Absolute (95% CI)** |  |  |
| **MDI at 6 months of age ( zinc with iron versus iron) (assessed with: Bayley (BSID-II))** | | | | | | | | | | | | |
| 2 | randomised trials | serious ^a^ | not serious | not serious | not serious | none | 180 | 179 | - | SMD **0.09 SD higher** (0.11 lower to 0.3 higher) | ⨁⨁⨁◯ MODERATE |  |
| **PDI at 6 months of age ( zinc with iron versus iron) (assessed with: Bayley (BSID-II))** | | | | | | | | | | | | |
| 2 | randomised trials | serious ^b^ | not serious | not serious | not serious | none | 180 | 179 | - | SMD **0.07 SD higher** (0.14 lower to 0.28 higher) | ⨁⨁⨁◯ MODERATE |  |
| **MDI at 12 months of age ( zinc with iron versus iron) (assessed with: Bayley (BSID-II))** | | | | | | | | | | | | |
| 4 | randomised trials | serious ^c^ | not serious | not serious | not serious | none | 389 | 401 | - | SMD **0.03 SD lower** (0.17 lower to 0.11 higher) | ⨁⨁⨁◯ MODERATE |  |
| **PDI at 12 months of age ( zinc with iron versus iron) (assessed with: Bayley (BSID-II))** | | | | | | | | | | | | |
| 4 | randomised trials | serious ^d^ | not serious | not serious | not serious | none | 389 | 401 | - | SMD **0.01 SD higher** (0.24 lower to 0.26 higher) | ⨁⨁⨁◯ MODERATE |  |

**CI:** Confidence interval; **SMD:** Standardised mean difference

#### Explanations

a. Two studies have unclear risk of bias.

b. Two studies have unclear risk of bias.

c. Two studies have unclear risk of bias and one study has high risk of bias.

d. Two studies have unclear risk of bias and one study has high risk of bias.

**Figure 1:** Funnel plot of comparison Zinc alone versus Placebo, outcome: MDI at 6 months of age


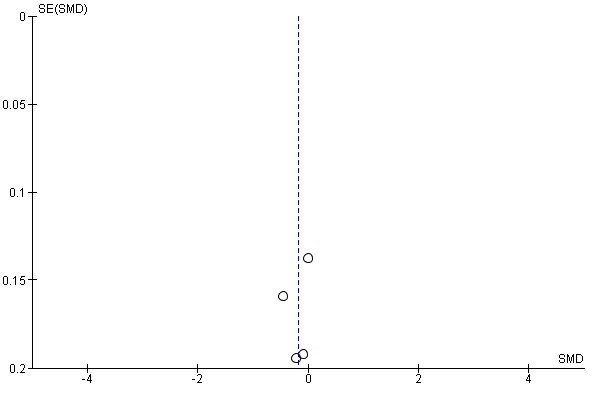


**Figure** **2:** Funnel plot of comparison Zinc alone versus Placebo, outcome: PDI at 6 months of age


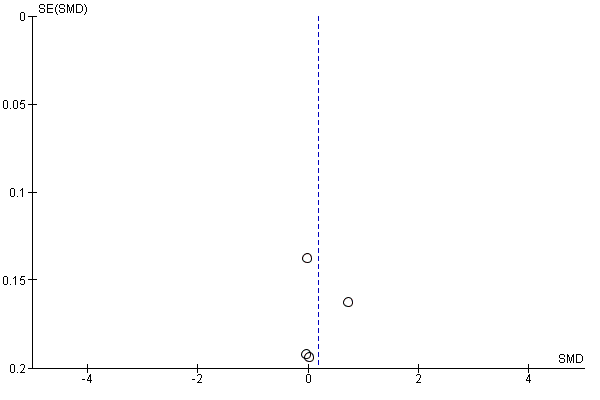


**Figure** **3:** Funnel plot of comparison Zinc alone versus Placebo, outcome: MDI at 12 months of age


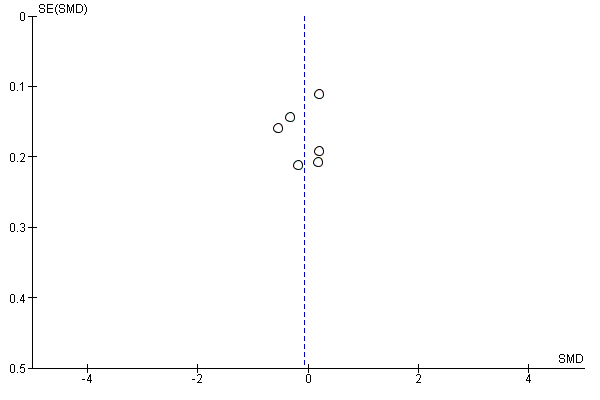


**Figure 4:** Funnel plot of comparison Zinc alone versus Placebo, outcome: PDI at 12 months of age


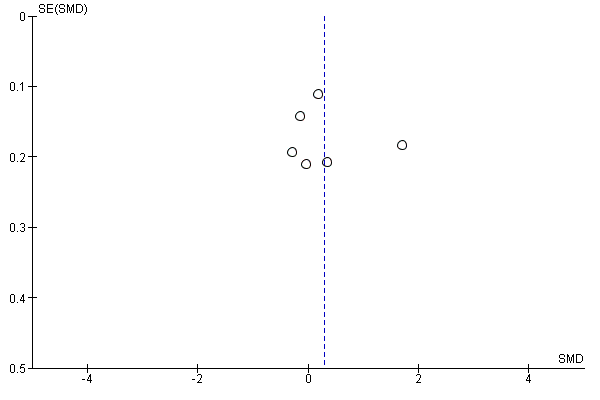


**Figure** **5:** Funnel plot of comparison Zinc with Iron versus Iron, outcome: MDI at 6 months of age


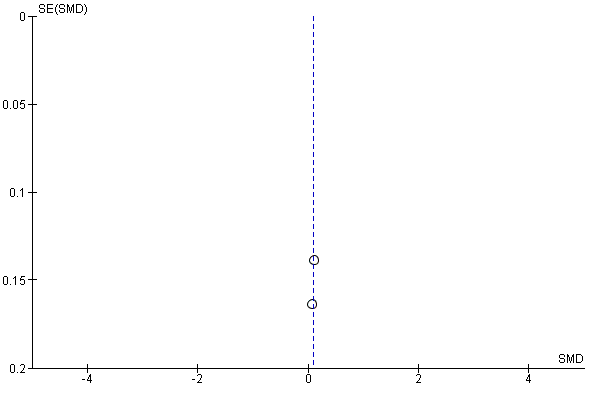


**Figure** **6:** Funnel plot of comparison Zinc with Iron versus Iron, outcome: PDI at 6 months of age


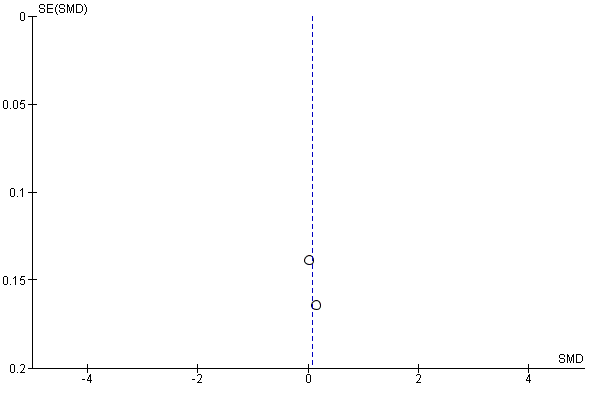


**Figure** **7:** Funnel plot of comparison: Zinc with Iron versus Iron, outcome: MDI at 12 months of age


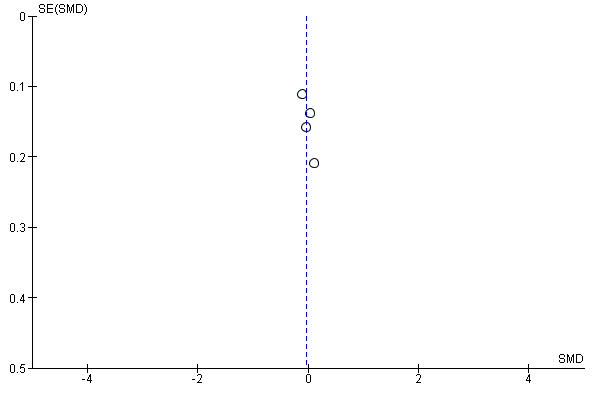


**Figure** **8:** Funnel plot of comparison: Zinc with Iron versus Iron, outcome: PDI at 12 months of age


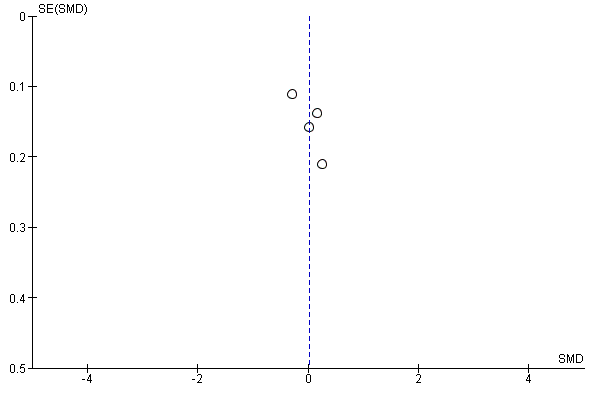


**Eggers Test P-values:**

1. MDI 6months Zinc alone: 0.22
2. PDI 6months Zinc alone: 0.32
3. MDI 12months Zinc alone: 0.67
4. PDI 12months Zinc alone: 0.12
5. MDI 6Months Zinc+ Iron: 0.17
6. PDI 6Months Zinc+ Iron: 0.22
7. MDI 12 Months Zinc+ Iron: 0.43
8. PDI 12 Months Zinc+ Iron: 0.2
